# Exosomes as therapeutic and drug delivery vehicle for neurodegenerative diseases

**DOI:** 10.1186/s12951-024-02681-4

**Published:** 2024-08-02

**Authors:** Zeinab Nouri, Ashkan Barfar, Sahra Perseh, Hamidreza Motasadizadeh, Samane Maghsoudian, Yousef Fatahi, Keyvan Nouri, Mohaddese Pourashory Yektakasmaei, Rassoul Dinarvand, Fatemeh Atyabi

**Affiliations:** 1 Department of Pharmaceutics, Faculty of Pharmacy, Tehran University of Medical Sciences, Tehran, Iran; 2 Nanotechnology Research Centre, Faculty of Pharmacy, Tehran University of Medical Sciences, Tehran, Iran; 3 Student Research Committee, Faculty of Pharmacy, Tabriz University of Medical Sciences, Tabriz, Iran; 4 Department of Pharmaceutical Nanotechnology, Faculty of Pharmacy, Tehran University of Medical Sciences, Tehran, Iran; 5 Student Research Committee, School of Medicine, Isfahan University of Medical Sciences, Isfahan, Iran; 6 Faculty of veterinary medicine, Garmsar Branch, Islamic Azad University, Garmsar, Iran; 7 Leicester School of Pharmacy, De Montfort University, Leicester, UK; 8 Dental Research Center, Dentistry Research Institute, Tehran University of Medical Sciences, Tehran, Iran

**Keywords:** Exosome, Neurodegenerative diseases, Targeted drug delivery

## Abstract

Neurodegenerative disorders are complex, progressive, and life-threatening. They cause mortality and disability for millions of people worldwide. Appropriate treatment for neurodegenerative diseases (NDs) is still clinically lacking due to the presence of the blood-brain barrier (BBB). Developing an effective transport system that can cross the BBB and enhance the therapeutic effect of neuroprotective agents has been a major challenge for NDs. Exosomes are endogenous nano-sized vesicles that naturally carry biomolecular cargoes. Many studies have indicated that exosome content, particularly microRNAs (miRNAs), possess biological activities by targeting several signaling pathways involved in apoptosis, inflammation, autophagy, and oxidative stress. Exosome content can influence cellular function in healthy or pathological ways. Furthermore, since exosomes reflect the features of the parental cells, their cargoes offer opportunities for early diagnosis and therapeutic intervention of diseases. Exosomes have unique characteristics that make them ideal for delivering drugs directly to the brain. These characteristics include the ability to pass through the BBB, biocompatibility, stability, and innate targeting properties. This review emphasizes the role of exosomes in alleviating NDs and discusses the associated signaling pathways and molecular mechanisms. Furthermore, the unique biological features of exosomes, making them a promising natural transporter for delivering various medications to the brain to combat several NDs, are also discussed.

## Introduction

Degenerative brain conditions encompass a variety of debilitating and diverse disorders characterized by progressive damage to neurons, leading to disruptions in their structure and function. The exact causes of neurodegenerative diseases (NDs) are still unclear, and no known effective treatments exist for these diseases. Among the different types of NDs, Parkinson’s disease (PD), Alzheimer’s disease (AD), multiple sclerosis (MS), stroke, and spinal cord injury (SCI) are highly prevalent. AD, as the most common form of dementia, affects more than 50 million patients age 65 and older. In 2016, the prevalence of PD was 6.1 million people worldwide, which is estimated to double by 2050 [1]. The prevalence of MS has increased worldwide in recent decades. The highest prevalence of MS is observed in Europe [2]. Stroke remains a leading cause of death and disability globally, accounting for over 11% of all deaths in 2019 [3]. The prevalence of SCI varies in different regions, with higher rates in low-income countries [4].

Most drugs designed to affect the brain often encounter difficulties in crossing the BBB successfully [5]. Numerous nanostructure formulations have been investigated to overcome the BBB, enhance drug effectiveness, reduce toxicity, increase specificity, and extend the circulation time of therapeutic drugs. Mainly, polymer-based nanoparticles, polymeric micelles, and liposomes, which are biocompatible and biodegradable, have gained significant attention [6]. However, despite their potential as delivery carriers, these nanostructures face challenges such as instability, rapid phagocytosis, and increased toxicity [7, 8].

Exosomes, which are tiny extracellular vesicles, are produced by many different cell types [9]. They have a round shape and small size (30–100 nm) and comprise a double-layered phospholipid membrane containing lipids, proteins, and nucleic acids. Exosomes can transmit necessary biological signals, affecting the body’s overall physiological state and cell communication. Furthermore, exosomes can impact synaptic plasticity, immunomodulation, angiogenesis, and neurogenesis [10, 11]. The cargoes carried by exosomes participate in crucial biological functions like cell signaling, nervous system development, maturation, apoptosis modulation, inflammation, autophagy, and oxidative stress [12–14].

Various critical applications of exosome and exosome-like particles have been extensively investigated. Exosomes have shown therapeutic potential in treating several diseases, including cancer, respiratory disease, degenerative diseases, and cardiovascular disease. Exosomes derived from stem cells can promote tissue repair and regeneration. Exosomes from human-induced pluripotent stem cells-derived mesenchymal stem cells (MSCs) promoted wound healing by activating collagen synthesis and stimulated angiogenesis [15]. Evidence has demonstrated that exosomes regulate immune responses and can be utilized as potential immunotherapeutic agents. They can potentially be used for antigen presentation, immune cell stimulation, and immunomodulation in cancer immunotherapy and autoimmune disorders. Panpan Ji et al. used a type of intelligent exosomes in which the exosome surface was modified with CD62L (L-selectin as a marker for tumor-draining lymph nodes (TDLNs)) and OX40L (a gene for activating T-cells and inhibiting regulatory T-cells). The engineered exosomes were specifically delivered to TDLNs. Furthermore, the smart exosomes suppressed T-reg cells and thereby augmenting the antitumor immune response and mitigating tumor development [16]. In the context of cardiovascular diseases, exosomes derived from human umbilical cord MSCs incorporated into hydrogel could successfully repair myocardial injuries after myocardial infarction [17]. Furthermore, MSC-exosomes exhibited therapeutic effects against severe steroid-resistant asthma via the regulation of macrophage polarization and inhibition of inflammation [18].

Exosomes have become a topic of great interest in studying degenerative neurological conditions. Exosomes have the potential to carry external substances, keep them in circulation, enter target cells, and release therapeutic agents, making them attractive natural carriers [19–21]. Moreover, they can breach the BBB easily, thereby creating a link between the central and peripheral compartments. Exosomes can evade immune responses and resist degradation by the body’s enzymes and RNases due to the protective effects of their lipid vesicles [22]. The significant role of exosomes in developing, diagnosing, and treating neurological disorders has been highlighted in various publications [23, 24]. Additionally, several publications have outlined the potential use of exosomes as a drug delivery system for brain cancers and related disorders [25–28]. Currently, There is no comprehensive review that explores the potential use of exosomes as a therapeutic and drug delivery vehicle for NDs.

This review aims to investigate potential therapeutic effect of exosomes by targeting signaling pathways involved in autophagy, apoptosis, inflammation, and oxidative stress. Furthermore, the involvement of exosomes in alleviating several types of neurodegenerative disorders, including PD, AD, MS, stroke, and SCI is discussed. Additionally, we elaborate on the possible application of exosomes for transporting therapeutic agents to the brain.

## Exosome composition and structure

**Fig. 1:**
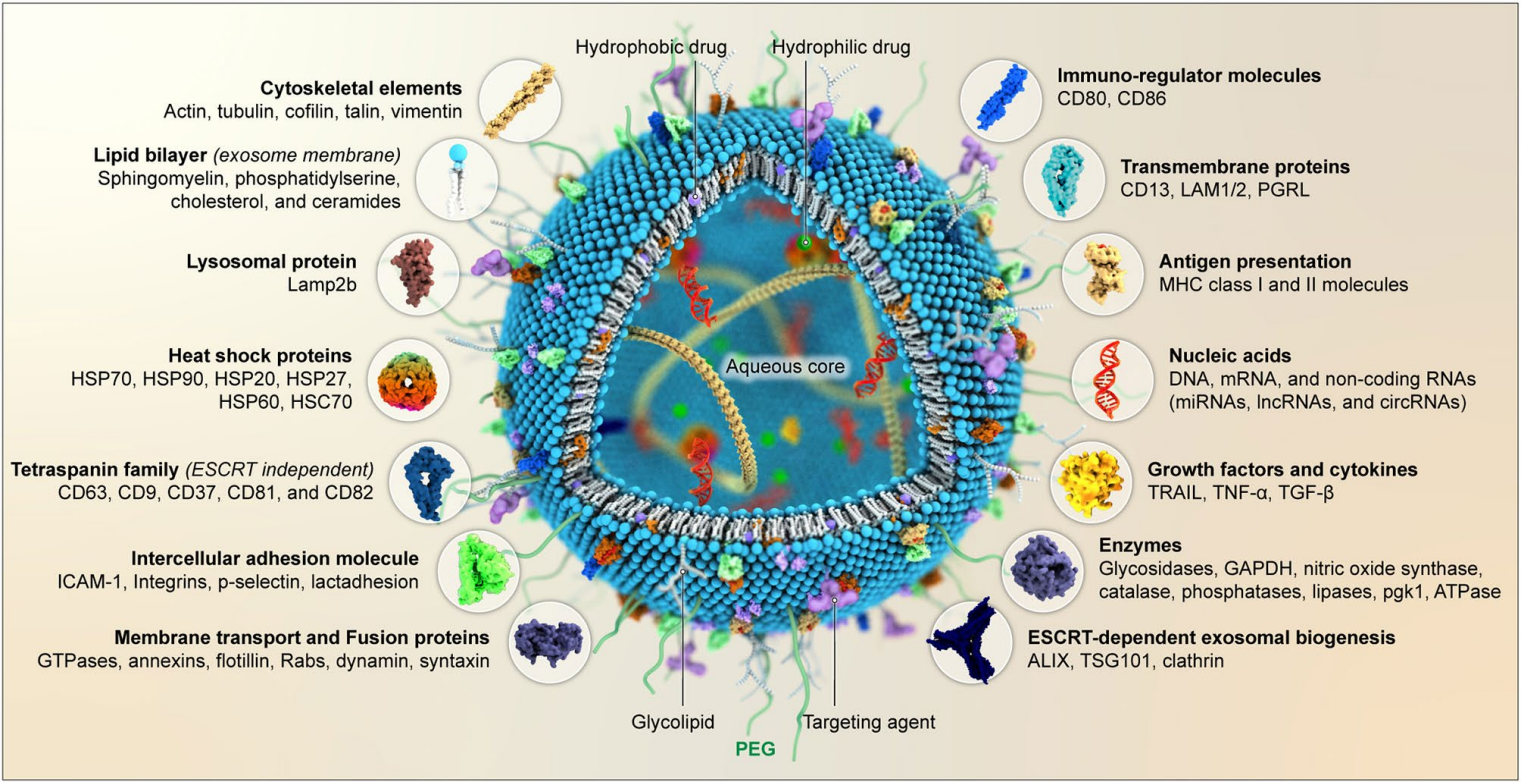
Schematic presentation of exosome structure and composition. CD, Cluster of differentiation; HSP, Heat shock proteins; ICAM, Intercellular adhesion molecule; TSG101, Tumor susceptibility gene 101; GAPDH, Glyceraldehyde-3-phosphate dehydrogenase; TGF–β, Transforming growth factor β; TNF-α, Tumor necrosis factor α; pgk1, Phosphoglycerate kinase; TRAIL, TNF-related apoptosis-inducing ligand; MHC, Major histocompatibility complex; Lamp, Lysosomal associated membrane protein

The structure of exosomes involves a lipid bilayer that encloses a central aqueous core. Their amphiphilic nature enables them to assimilate hydrophilic and lipophilic molecules, making them valuable in medication administration [29]. Exosomes have a heterogeneous composition, which varies depending on their origin and physiological state. As illustrated in Fig. 1, exosomes are rich in specific lipids, proteins, enzymes, and genetic materials that participate in intercellular interaction and transport signaling molecules to nearby and distant locations [30]. Various lipid derivatives such as sphingomyelin, phosphatidylserine, cholesterol, and ceramides are commonly found in exosome membranes [31]. Analyzing exosome composition revealed the presence of common proteins such as tetraspanins, cytoskeletal elements, lysosomal proteins, enzymes, membrane transport and fusion proteins (GTPases, annexins, and flotillin), antigen presentation molecules (Major histocompatibility complex (MHC) class I and II), and intercellular adhesion molecule (ICAM-1) [32].

Exosomes encompass heat shock proteins (HSP) such as HSP70 and HSP90, contributing to displaying antigens. Additionally, exosomes contain an abundant group of proteins called tetraspanins. These transmembrane proteins can interact with various partners, such as MHC molecules and integrins, indicating their significant involvement in forming complex molecular structures [33].

Exosomes are enriched with specific proteins, such as ALIX and TSG101, that are vital for the biogenesis of exosomes through the endosomal sorting complex required for transport (ESCRT). Additionally, tetraspanin proteins, including CD63, CD9, CD37, CD81, and CD82, have also been discovered to be involved in an ESCRT-independent pathway [24]. Typically referred to as typical exosome markers, these proteins are distinctively absent in other groups of vesicles [34].

Furthermore, alongside the proteins typically present in exosomes, certain proteins in exosomes are exclusive to the specific cellular origin. For instance, exosomes derived from intestinal epithelial cells possess distinct proteins such as syntaxin 3, C26, and A33, which are dependent on the side of the epithelial membrane for secretion, either apical or basolateral [35]. Moreover, other examples of cell-specific exosomes include α4β1 on reticulocytes and platelets expressing P selectin [36].

In addition, RNA sequencing techniques have shown that exosomes transport a diverse range of genetic material, like DNA, mRNA, and various types of non-coding RNAs such as circular RNAs (circRNAs), miRNAs, and long non-coding RNAs (lncRNAs). lncRNAs contribute to controlling cell differentiation and modulating the cell cycle, while circRNAs have been proposed as suppressors of miRNAs, competing with them to regulate gene expression [36].

## The potential therapeutic effect of exosomes

Exosomes can transport proteins and RNAs to recipient cells. These cargoes can trigger or inhibit various signaling pathways involved in inflammatory processes, apoptosis, autophagy, and oxidative stress. As a result, exosomes can potentially contribute to the alleviation or advancement of multiple diseases.

**Fig. 2:**
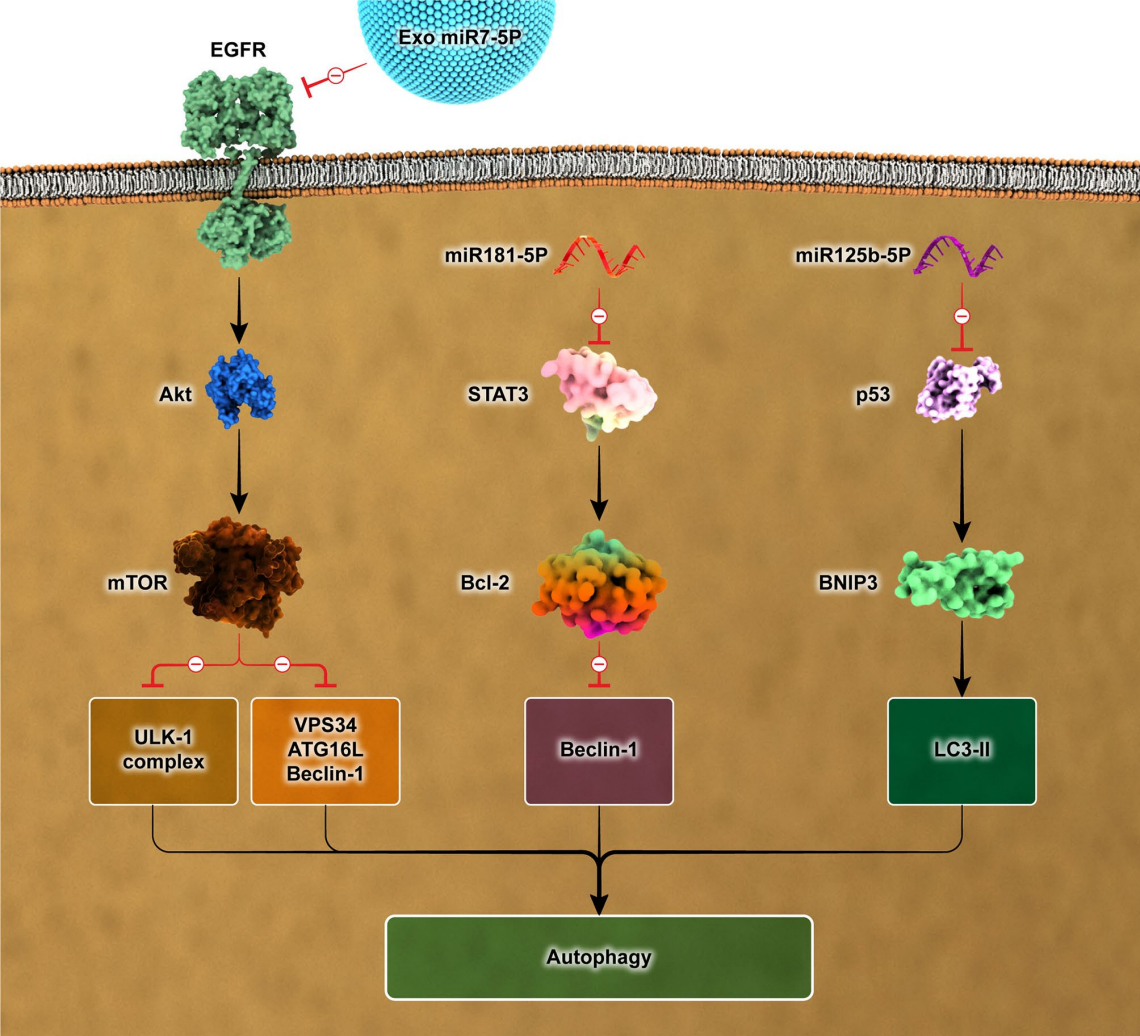
Exosomes and exosomes-derived miRNAs can interface several signaling pathways involved in autophagy. Bcl-2, B-cell lymphoma 2; BNIP, BCL2/adenovirus E1B 19 kd-interacting protein; mTOR, Mammalian target of rapamycin; EGFR, Epidermal growth factor receptor; STAT, Signal transducer and activator of transcription; ULK, Unc-51 like autophagy activating kinase; VPS34, Vacuolar protein-sorting 34; ATG, Autophagy-related protein; LC3, Light chain 3

Autophagy involves pathways that recycle unnecessary and dysfunctional parts of the cytoplasm. The formation of autophagosomes is a crucial aspect of this procedure [37]. Current research has suggested a connection between exosomes and autophagy pathways (Fig. 2), which can impact diseases like cardiovascular, liver, and kidney diseases. The influence of exosomes on autophagy can occur through two mechanisms: the direct transfer of cargoes into the cytoplasm and the interaction with specific receptors like epidermal growth factor receptor (EGFR) and TLR1/2/4/6, which are present on the cellular plasma membrane [38].

Numerous studies have extensively demonstrated the role of TLR2/6 ligands in triggering autophagy. As a result, exosomes derived from neutrophils infected with mycobacterium tuberculosis contain TLR2/6 ligands that induce autophagy in macrophages. This mechanism significantly contributes to the elimination of mycobacterium tuberculosis [39]. Autophagy is regulated by EGFR induction through the activation of several cellular signaling pathways, including PI3K/AKT/mTOR, RAS/MAPK, and JAK/STAT [40]. The elevated levels of autophagic markers, specifically LC3II, in human bronchial epithelial cells suggested an enhancement of autophagy caused by exosomal miR-7-5p. This enhancement was achieved by directing attention to the EGFR/Akt/mTOR signaling pathway [41]. Moreover, MSC-secreted exosomes demonstrated the ability to improve cardiac function and reverse autophagy induced by ischemia by inhibiting the p53/Bnip3 signaling pathway. The authors propose that the existence of miR-125b-5p within these exosomes contributes to their therapeutic benefits [42].

Multiple studies have suggested that the PI3K/AKT, MAPK, and AMPK pathways are critical in autophagy activation, with mTOR acting as a suppressor of this process [43]. Exosomes originating from MSCs have demonstrated the capacity to improve cardiac repair by inhibiting apoptosis and enhancing autophagy. The activation of autophagy is linked with the AMPK/mTOR and AKT/mTOR pathways [44]. An additional study revealed that exosomes obtained from MSCs significantly impacted the concentration of autophagic markers, such as LC3-II and Beclin-1, by disrupting the mTOR signaling pathway. This disruption ultimately resulted in the alleviation of diabetic kidney damage [45]. Additionally, miR-486 in ADSCs-exosomes activated autophagy by blocking the podocytes’ Smad1/mTOR signaling pathway. This finding presents a potentially hopeful approach to treating diabetic nephropathy [46].

Multiple studies have shown the critical importance of STAT3 in inhibiting autophagy. Downstream of STAT3, Bcl-2 interacts with Beclin1 to regulate autophagy [47]. The presence of exosomes containing miR-181-5p has been found to promote autophagy in liver fibrosis by inhibiting the STAT3/Bcl-2/Beclin1 pathway, resulting in reduced liver fibrosis [48].

**Fig. 3:**
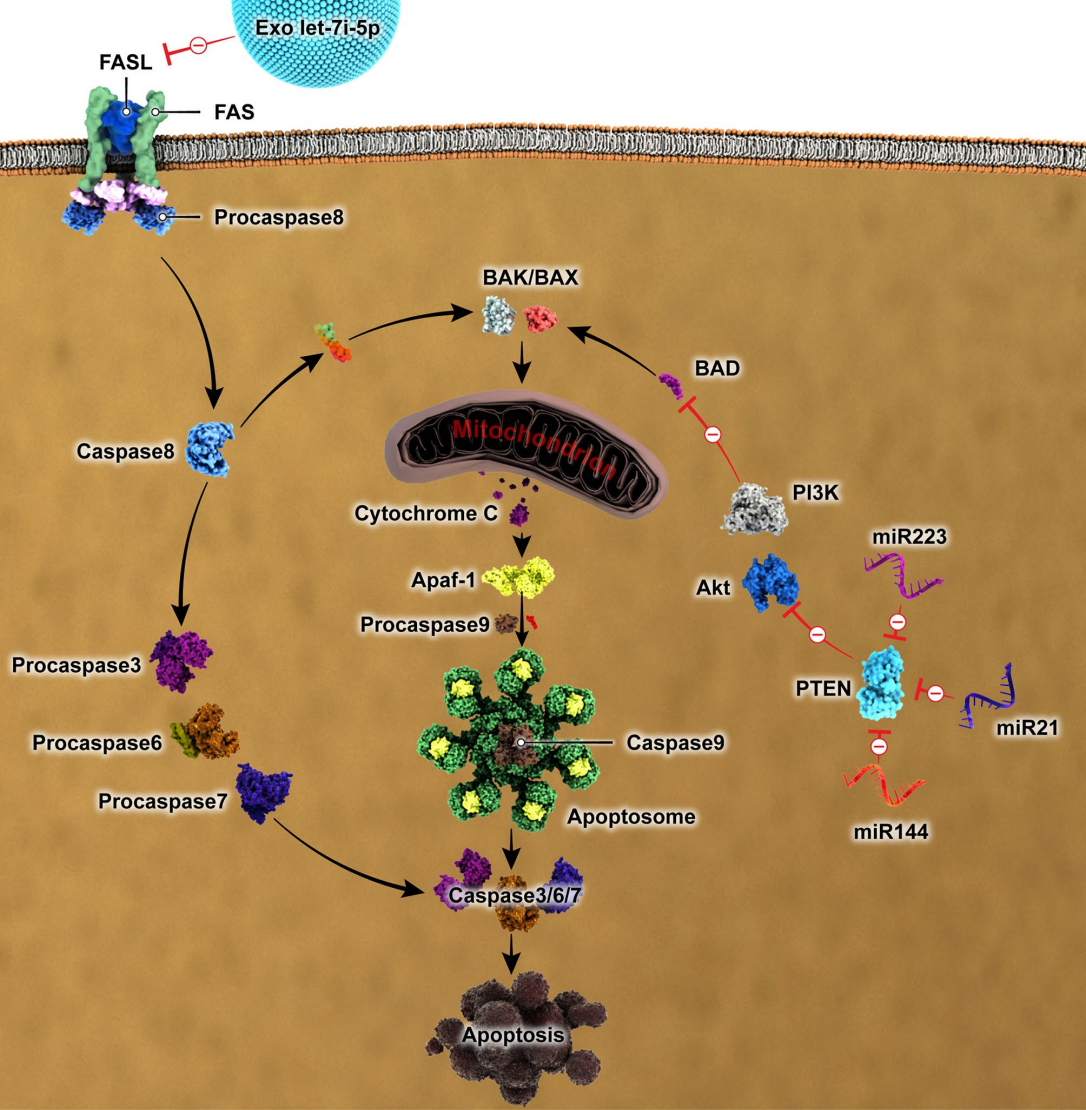
Main signaling pathways involved in apoptosis influenced by exosomes and exosomes-derived miRNAs. PI3K/AKT, Phosphatidylinositol-3 kinase/AKT; PTEN, Phosphatase and tensin homolog; BAX, Bcl-2-associated X protein; Apaf-1, Apoptotic protease activating factor-1; FASL, Fas ligand; miR, microRNA

The role of apoptosis in advancing several diseases and their consequential detrimental effects are widely acknowledged. The significant contribution of miRNAs encapsulated within exosomes to the attenuation of apoptosis has been extensively documented (Fig. 3). Exosomes made from human umbilical cord MSCs were able to stop apoptosis and inhibit LC3B-II/I and beclin1 from being expressed in H9C2 cells. The activation of the PI3K/AKT/mTOR signaling pathway was found to be the fundamental mechanism [49]. Exosomal miRNAs protect against intestinal damage by inhibiting inflammation and reducing apoptosis in lipopolysaccharide (LPS) presence. The suppressive effects of milk exosomes on the TLR4/NF-κB and p53 pathways were found to be associated with their capacity to diminish inflammation and prevent cell death [50]. In addition, exosomal miR-21 was found to have an anti-apoptotic impact when faced with cell death induced by oxidative stress in myocardial cells H9C2 by downregulating programmed cell death 4 (PDCD4) [51]. PDCD4 is recognized for encouraging tumor cell death and inhibiting tumor metastasis [52]. Furthermore, research has demonstrated that phosphorylated p38 plays a pivotal function in inducing cell death by activating critical proteins involved in apoptosis. Exosomes originating from MSCs were found as carriers of miR-21 and alleviated cell death caused by hypoxia in beta cells by suppressing p38 MAPK signaling [53].

Zhang et al. discovered that exosomal miRNAs, specifically miR-152-3p and let-7i-5p, effectively prevented apoptosis stimulated by hypoxia in H9C2 cells. They observed that miR-152-3p hindered the expression of Atg12, a protein involved in intrinsic apoptosis that regulate apoptosis by reducing the activity of anti-apoptotic members within the Bcl-2 family [54, 55]. Furthermore, let-7i-5p reduced Fasl level, a gene encoding Fas ligand (FASL) from the tumor necrosis factor family, which participates in the extrinsic apoptotic pathway through engaging the death receptor pathway [56].

The downregulation of phosphatase and tensin homolog (PTEN) expression by exosomal miR-144 can suppress the PI3K/AKT signaling pathway and enhance the expression of PI3K/AKT proteins, leading to the inhibition of cardiomyocyte apoptosis under hypoxic conditions [57]. Similarly, isolated miR-223 from exosomes originating from MSCs showed an anti-apoptotic effect on neurons by inhibiting PTEN, initiating the PI3K/AKT pathway [58]. Moreover, it was observed that exosomal miR-106a-3p attaches to CASP9, impeding the pathway associated with caspase signaling in human vascular smooth muscle cells, suggesting its promise as a therapeutic intervention for atherosclerosis treatment. Additionally, exosomes derived from induced pluripotent stem cells have been confirmed to enhance the survival of ischemic cardiomyocytes and protect them from oxidative damage by suppressing apoptosis. The beneficial effects of exosomes are likely due to the presence of two significant miRNAs, specifically miR-21 and miR-210 [59].

**Fig. 4:**
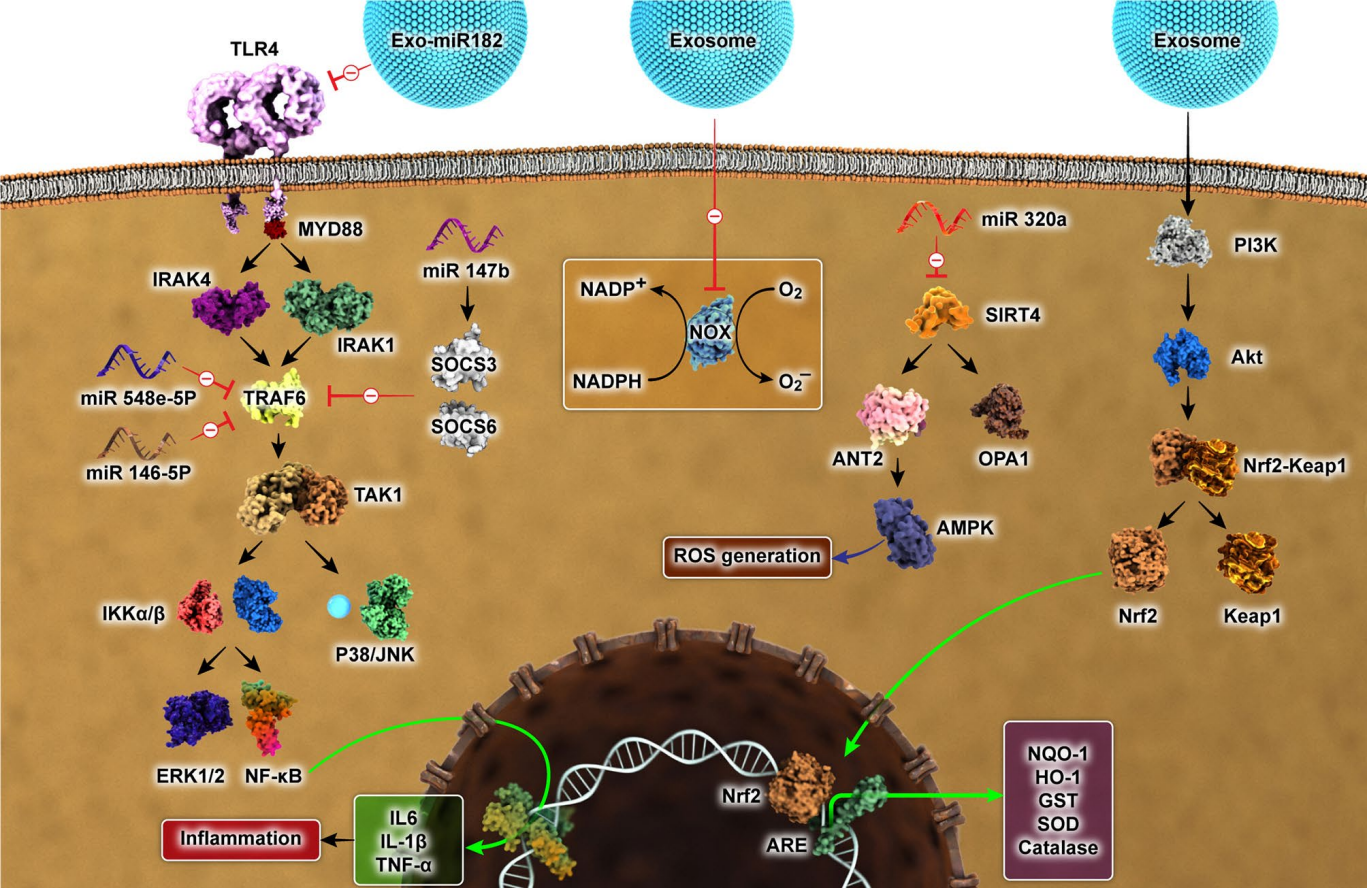
Numerous therapeutic targets affected by exosomes and exosomes-derived miRNAs toward modulating inflammation and oxidative stress. AMPK, (AMP)-activated protein kinase; PI3K/AKT, Phosphatidylinositol-3 kinase/AKT; Nrf2/ARE, Nuclear factor E2-related factor 2/ antioxidant response element; SIRT, Sirtuin; OPA1, Optic atrophy 1; ANT2, Adenine nucleotide translocator 2; SOD, Superoxide dismutase; HO-1, Hemoxygenase-1; NQO-1, NAD(P)H quinone dehydrogenase1; GST, Glutathione S-transferase; IL, Lnterleukin; TNF-α, Tumor Necrosis Factor-α; SOCS, Suppressor of cytokine signaling; ERK, Extracellular-signal-regulated kinase; JNK, c-Jun N terminal kinase; NF-κB, Nuclear factor-κB; IRAK, Interleukin-1 receptor-associated kinase; TRAF, Tumor necrosis factor receptor-associated factor; TLR, Toll-like receptor; MyD88, Myeloid differentiation factor 88; TAK, Transforming Growth Factor (TGF)-β-activated Kinase; NADPH, Nicotinamide adenine dinucleotide phosphate; IKK, Ikappa kinase

Multiple studies have shown that exosomes regulate immune cells, cytokines, and inflammatory mediators in the inflammatory microenvironment (Fig. 4). M1 and M2 are the two main classifications of macrophages. M1 macrophages play a role in the formation of inflammatory conditions through the secretion of pro-inflammatory factors. Conversely, M2 macrophages stimulate the release of anti-inflammatory factors such as interleukin-10 (IL-10), transforming growth factor beta (TGF-β), C-C motif chemokine ligand 1 (CCL1), and CCL17 [60]. A study has shown that exosomes derived from MSCs, which carry miR-182, can hinder the TLR4/NF-κB pathway and enhance the PI3K/AKT pathway, resulting in an accelerated polarization of M2 macrophages in cardiac ischemia/reperfusion (I/R) damage [61]. Similarly, exosomes derived from human placental MSCs have been shown to inhibit inflammation in the RAW264.7 macrophage cell line by targeting the TLR4-associated NF-κB/MAPK and PI3K signaling pathways [62]. Exosomal miRNAs obtained from MSCs generated from amniotic fluid (AF), including miR-146a-5p and miR-548e-5p, were found to suppress the proteins linked to the NF-κB pathway, namely TRAF6, during exposure to LPS. Additionally, AF-MSCs-exosomes assisted with reducing the activation of mitogen-activated protein kinase components (JNK, ERK1/2, and P38) triggered by LPS [63]. These findings suggest that exosomal miR-146a-5p and miR-548e-5p hold promise as therapeutic interventions for addressing excessive inflammation-induced preterm birth. Exosomes isolated from IL-1b-primed MSCs relieved inflammation in osteoarthritic SW982 cells. miR-147b was more expressed in MSC-IL-Exosomes than MSC-Exosomes and, therefore, revealed higher inhibitory outcomes on the expression of the pro-inflammatory factors than MSC-Exosomes. In addition, MSCs-IL-Exo increased the levels of anti-inflammatory elements (SOCS3 and SOCS6) [64].

Oxidative stress refers to the condition characterized by disruption of redox homeostasis. This phenomenon changes how the body responds to inflammation, autophagy, and programmed cell death, and it also plays a role in the oxidation of nucleic acids, proteins, and lipids [65]. Nrf2, also known as nuclear factor erythroid 2-related factor 2, plays a crucial role in maintaining the balance of oxidation and reduction in cells. In the presence of oxidative stress, Nrf2 translocates to the cell’s nucleus. It initiates a cascade of reactions that activate antioxidant enzymes like NAD(P)H quinone oxidoreductase 1 (NQO1) and heme oxygenase 1 (HO-1) to modulate cellular oxidative stress. Compelling studies have indicated that exosomes can abolish oxidative insult in target cells by transferring miRNA and antioxidative enzyme mRNA or proteins (Fig. 4).

According to a recent report, researchers have discovered that exosomal miR-320a suppresses SIRT4 and its associated proteins (ANT2, AMPK, and L-OPA1) in an experimental mouse model simulating early ovarian insufficiency. This inhibitory function is crucial in preventing the development of reactive oxygen species (ROS) 204. Multiple studies have shown that the PI3K/Akt/eNOS pathway reduces oxidative stress [66]. Research has shown that miR-132-3p enhances the effects of MSC-exosomes by activating the Ras/PI3K/Akt/eNOS signaling pathway in microvascular endothelial cells damaged by hypoxia/reoxygenation (H/R) [67]. In another investigation, MSC-derived exosomes contained antioxidant miRNAs that have a significant antioxidant effect, as evidenced by an increased function of enzymes like catalase, superoxide dismutase (SOD), and glutathione peroxidase. They reduced ROS production and decreased damage to DNA, lipids, and proteins in preclinical models simulating injury triggered by seizures. These antioxidant properties of MSC-exosomes are believed to be facilitated via the Nrf2 signaling pathway [68].

Exosome obtained from Adipose-derived stem cells (ADSCs) has been observed to hinder ROS production and reduce the levels of cytokines induced by LPS, providing a protective effect against sepsis. These beneficial effects were consistent with reduced Keap1 expression, which acts as a suppressor of Nrf2 [69]. Exosomes obtained from umbilical cord MSCs have also been discovered to boost diabetic wound healing by mitigating NOX1 and NOX4 and promoting angiogenesis [70]. The Nrf2/NQO-1 pathway can be activated by exosomes from MSCs in the human umbilical cord to improve non-alcoholic steatohepatitis [71].

Overall, exosomes hold significant therapeutic potential for various diseases by targeting multiple signaling pathways involved in autophagy, apoptosis, oxidative damage, and inflammation. Although their promising effects have been demonstrated in cell culture systems, further in vivo investigations are needed to confirm their therapeutic applications.

## Exosomes as promising brain drug carriers

**Fig. 5:**
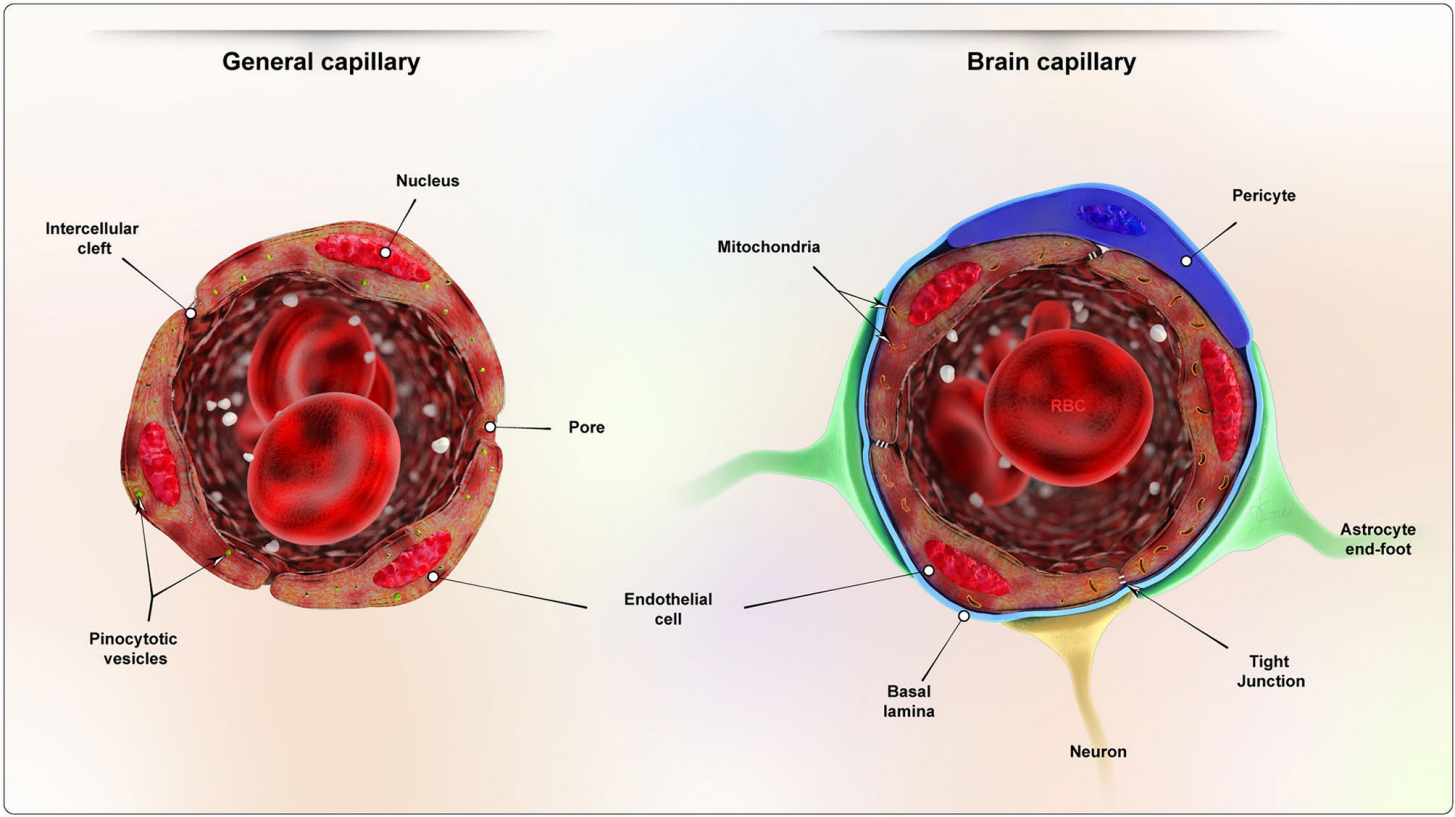
Structural difference between the cerebral and non-cerebral capillaries. Reprinted from Ref [73] with permission from John Wiley and Sons

BBB is known for its crucial role in effectively controlling the flow of potentially harmful substances from the blood to the brain [72]. This physiological barrier impeded the movement of various components to reach the brain and posed multiple difficulties for current drug delivery methods. The BBB is a complex structure consisting of several neurovascular units, such as brain capillary endothelial cells (BCECs), pericytes, basal lamina, astrocytes, microglia, and neural cells. There is a structural difference between the endothelium of cerebral and non-cerebral capillaries due to the presence of endothelial tight junctions (Fig. 5) [73]. The BBB affects large and hydrophilic molecules. It allows the passage of small, lipophilic molecules or those with fewer than nine hydrogen bonds, along with gases like carbon dioxide and oxygen [74]. Scientists have devised different nano-based carriers to surmount the BBB and target medications to specific locations within the brain. However, these nanoformulations have two significant obstacles: toxicity and rapid elimination of drugs by the reticuloendothelial system [75].

Exosomes have unique advantages compared to synthetic alternatives, such as liposomes and polymeric nanoparticles. These advantages include biocompatibility, stability, prolonged blood circulation, low immunogenicity, and low intrinsic toxicity. Additionally, due to the abundance of proteins and lipids on their membrane, exosomes exhibit a high capacity for uptake by specific target cells [76, 77]. Remarkably, exosomes can traverse or circumvent biological barriers such as BBB, even without surface modifications.

Certain cell-derived exosomes, like those from MSCs, neurons, macrophages, and endothelial cells, are particularly permeable through the BBB compared to exosomes from other cells [74]. Although it has been established that exosomes can traverse the BBB, the specific molecular mechanisms involved remain unknown.

The ability of exosomes to target specific cells is influenced by both the content they carry and the physiological condition of the recipient cells. In situations where there is neuroinflammation or nerve injury, exosomes can enter the brain. Specifically, exosomes originating from MSCs have been observed to accumulate in brain regions affected by inflammation and absorbed by glial cells rather than neuronal cells [78]. This implies that exosomes can be used for precise drug delivery to brain-related disorders. In a research inquiry, unmodified exosomes isolated from macrophages, which contained a brain-derived neurotrophic factor (BDNF) protein, demonstrated effective BBB penetration. This delivery was further enhanced by 3.6 times in the presence of neuroinflammation [79].

Noninvasive delivery of therapeutic agents to the brain can be achieved using different approaches, including receptor-mediated transcytosis (RMT) and adsorption-mediated transcytosis. Among these methods, RMT stands out as the most extensively researched and employed method for delivering drugs across the endothelial cells of the BBB. It can also be utilized to transport surface-modified exosomes to different areas of the brain. Using specific ligands that interact with RMT, such as the transferrin receptor, low-density lipoprotein receptor, and insulin receptor, it is feasible to elicit BBB crossing via RMT-mediated mechanisms [80]. T7, a transferrin receptor-binding peptide, could improve the effectiveness of exosomes as smart carriers for AMO-21, an antisense miRNA oligonucleotide targeting miR-21. This surface modification showed improved results in alleviating a rat model of glioblastoma compared to using unmodified exosomes [81].

Extensive research has focused on neurotropic viruses due to their capacity to penetrate brain tissue, suggesting the potential utilization of viruses or their constituents as transporters for delivering therapeutic substances to the brain. One example includes the utilization of peptides obtained from the glycoprotein of the rabies virus (RVG), which have shown promise in targeting the brain and traversing the BBB. To further enhance this approach, Yu and colleagues modified exosomes to present RVG peptides on their surface, specifically targeting a7-nAChR, a receptor present in the brain. These modified exosomes also contained a neprilysin variant that selectively degraded amyloid-β (Aβ), a key component in AD development. The formulated exosomes successfully reached and positioned themselves within the brain’s hippocampus region, effectively reducing Aβ40 levels and alleviating inflammation [82].

Collectively, exosomes promise to serve as advanced nanocarriers for addressing brain diseases, thanks to their inherent ability to circumvent the BBB, favorable physical characteristics, and the ability to modify their surface. Although exosomes possess desirable physical characteristics, multiple biotechnology strategies and engineered methods are still needed to enhance target recognition, facilitate efficient traversal of BBB, and achieve appropriate biodistribution. More extensive research is necessary to advance imaging techniques for tracking the transport of exosomes to the brain and to enhance our understanding of their traversal through the BBB.

## Role of exosomes and exosomes delivered cargoes in different types of NDs

### Ischemic stroke

An acute ischemic stroke occurs when there is a sudden disturbance in the blood flow to a particular region of the brain, leading to impaired neurological function. This condition usually occurs due to the blockage of a blood vessel in the brain, which can happen either as a result of a blood clot (thrombosis) or the formation of a clot that has traveled from another part of the body (embolism) [83]. During a vessel blockage, the brain experiences a central region where irreparable damage occurs and a surrounding area known as the penumbra, where brain function is compromised due to reduced blood flow but not yet irreversibly damaged [84].

Pei and colleagues utilized the ultracentrifugation method to obtain exosomes derived from astrocytes (AS-Exo). These AS-Exo exosomes have shown the ability to improve the survival of neurons and reduce apoptosis triggered by oxygen and glucose deprivation (OGD). Furthermore, in vivo experiments demonstrated the beneficial effect of AS-Exo exosomes on neuronal injuries by regulating autophagy [85]. In 2020, researchers discovered that exosomes sourced from brain endothelial cells augmented the BrdU/nestin-positive cells in the rats’ selected brain regions when directly administered into the ventricles [86]. Nestin is a protein exclusive to neuroepithelial stem cells and part of the cytoskeletal intermediate filament family. This protein is pivotal in regulating stem cell functions [87]. This finding highlights the potential of exosomes in regenerating vascular structures in neurons and safeguarding the brain during acute ischemic injury.

Additional research has discovered that brain endothelial exosomes can enhance motor function and augment blood flow in specific brain regions of mice that have experienced middle cerebral artery occlusion/reperfusion (MCAO/R) and related injuries. Furthermore, these exosomes have been observed to bolster the expression of synaptic regulatory proteins while safeguarding against brain cell death. Notably, associations have been observed between locomotor activity, spatial memory, neuronal activity, and concentrations of exosomes within the rat central nervous system (CNS) subjected to MCAO [88].

In the brain of rats that underwent MCAO, there was an increase in inflammation-related genes such as NF-κB, TNF-α, IL-6, iNOS, and COX2. Nevertheless, introducing exosomes derived from neural stem cells (NSCs) that express the tumor susceptibility gene 101 (TSG101) resulted in a significant reduction in the expression of the enzymes mentioned above. Previous studies have demonstrated that TSG101 and adapter molecules involved in signal transduction have a critical role in the creation and release of exosomes. Earlier research has shown that overexpressing TSG101 significantly increases the secretion of exosomes [89, 90].

In a stroke, diverse cellular mechanisms, encompassing cell migration, proliferation, differentiation, apoptosis, vascularization, and oxidative stress, are heavily influenced by the regulation of the PI3K/AKT pathway [91]. The in vitro OGD experiment results demonstrated a significant positive impact of exosomes on protecting primary cortical neurons in OGD-exposed rats. This research suggested that the observed protective effect could be attributed to the increased levels of BDNF and regulation of the PTEN/AKT pathway [92]. As mentioned previously, BDNF has shown effectiveness in treating CNS disorders, including stroke, by various mechanisms, and it holds significant potential for repairing brain damage. Additionally, combining BDNF with other stroke treatment strategies has been reported to be beneficial [93].

Intracellular investigations can be conducted to investigate the impact of exosomes on stroke when combined with BDNF. In a study examining I/R injury, exosomes derived from human embryonic kidney cells overexpressing BDNF were utilized. The BDNF-Exosomes therapy prevented apoptosis caused by H/R by reducing oxidative stress and calcium ion loss. It also maintained the stability of the mitochondrial membrane potential in damaged SY-5Y cells exposed to I/R [94].

Another research by Zhu et al. demonstrated that exosomes sourced from NSCs incorporating BDNF significantly reduced levels of BAX and cleaved caspase 3 when exposed to H_2_O_2_, indicating a decrease in cellular apoptosis. Moreover, engineered exosomes exhibited an activity that facilitated the transformation of NSCs into neurons [95].

The excessive presence of ROS is commonly observed during ischemic injury, particularly in cases of ischemic stroke [96]. One potential solution to mitigate this issue is the utilization of edaravone (EDV), a potent scavenger of free radicals. Administration of exosomes and EDV via intravenous injection in mice has revealed positive effects on neurological function during the early stages of stroke. This is attributed to the brain-specific targeting mechanism enabled by the interaction of transferrin with its corresponding receptor. Furthermore, Exosomes containing EDV have exhibited neuroprotective properties in the permanent MCAO model and facilitate the transition of microglia polarization from M1 to M2. [97, 98].

Exosomal-mediated drug delivery allows neuroprotective drugs to transport across the BBB, making them suitable drug carriers for NDs. Huang and colleagues demonstrated that Baicalin loaded in exosomes (Exo-Baicalin) exhibits increased solubility and enhanced brain targeting compared to Baicalin alone in both transient MCAO and pMCAO models in mice. Moreover, Exo-Baicalin effectively inhibits ROS generation and activates the Nrf2/HO-1 pathway [99]. Exosomes derived from embryonic stem cells and loaded with curcumin (Cur) provided additional benefits compared to exosomes or Cur alone. These advantages stem from the combination of the anti-inflammatory properties of exosomes and the antioxidant effects of Cur. Cur-exosomes displayed promising therapeutic effects in mitigating I/R injury by suppressing the buildup of ROS in brain lesions and reducing apoptosis associated with mitochondria. In addition, Cur–exosomes exhibited more solubility, stability, and bioavailability than Cur alone [100]. Quercetin (Que) was incorporated into surface-modified exosomes and conjugated with a monoclonal antibody against growth-associated protein 43 (GAP43) to target the impaired neurons and enhance their survival. This conjugation increased the accumulation of Que in the damaged brain region and allowed for the blocking of ROS generation by targeting the Nrf2/HO-1 pathway [101].

The increased expression of nicotinamide phosphoribosyltransferase (Nampt) is widely acknowledged to significantly improve cerebral injuries caused by ischemia. Exosomes derived from astrocytes could restore neuronal damage and boost neuronal autophagy caused by OGD/R. The secretion of Nampt by these exosomes enhanced autophagy and suppressed apoptosis by interfering with the AMPK/mTOR pathway [102].

Recent evidence has indicated that pigment epithelium-derived factor (PEDF) possesses neuroprotective and anti-inflammatory characteristics [103]. Exosomes containing PEDF have been found to alleviate injury caused by I/R by promoting autophagy and preventing apoptosis [104]. Furthermore, increasing evidence suggests Zeb2/Axin2’s role in neurogenesis contributes to post-stroke functional recovery [105]. Exosomes delivering Zeb2/Axin2 have been shown to enhance post-stroke neuroplasticity, increase neurogenesis, and boost the concentration of BDNF [106].

Exosomes have a critical function in transferring miRNAs across the BBB membrane. There are two different strategies for using exosomal miRNAs to alleviate CNS diseases. One approach involves using exosomes containing miRNAs, while another consists in incorporating the exogenous miRNAs into exosomes that hinder disease-mediated genes or fortifying neuroprotective factors [107].

Following MCAO, the concentration of miR-210, a microRNA, is initially elevated at the injury site. However, within the first hour up to 24 h post-MCAO, there is a significant decrease in miR-210 concentration. During this critical period, RGD (Arg-Gly-Asp) -exosomes loaded with miR-210 has enhanced miR-210 expression, leading to increased levels of angiogenic factors and improved animal survival rates [108].

The transfer of miR-21-5p through BMSC-Exosomes alleviated ischemic brain damage and accelerated angiogenesis. This effect was evidenced by the upregulation of expression levels of vascular endothelial growth factor (VEGF), VEGF Receptor 2 (VEGFR2), angiopoietin-1 (Ang-1), and tyrosine kinase with immunoglobulin-like and EGF-like domains 2 (Tie-2) [109]. MiR-22-3p, present in exosomes sourced from adipose-derived MSCs (ADMSCs), mitigates ischemic brain injury by modulating the BMF/BMP2 axis, inhibiting the actions of KDM6B. Studies have indicated that elevated BMF levels can accelerate neuronal death, and KDM6B contributes to ischemic injury by upregulating BMF through BMP2 [110].

Urine-derived stem cell exosomes enhanced neuron generation and reduced neurological impairments in post-ischemic stroke rats, and in vitro experiments with neural stem cells exposed to OGD/R revealed that exosomes stimulated the growth and differentiation of neurons, possibly by transferring exosomal miR-26a, which inhibits the expression of histone deacetylase 6 (HDAC6). The findings suggest that exosomes have the potential to be an effective therapeutic strategy for treating brain ischemia [111]. In a study conducted in 2021, in vitro trials indicate that exosomes originating from mesenchymal cells of the umbilical cord (hUMSC-Exo) reduced inflammation in microglia following OGD. In vivo investigations revealed a notable decrease in infarct size and reversed behavioral abnormalities in mice treated with exosomes containing miR-146a–5a, which functioned by blocking the inflammatory response through the IRAK1/TRAF6 pathway. This pathway also reduces the pro-inflammatory actions of microglia [112].

Administering melatonin to rats has been found to trigger the production of plasma exosomes, exhibiting a protective effect against inflammation and cellular demise caused by inflammasomes in the context of reduced blood flow. The administration of melatonin-treated exosomes has demonstrated diminished tissue harm and enhanced functional recovery by modulating the toll-like receptor 4 (TLR4)/NF-κB signaling pathway. Additionally, melatonin administration provoked changes in the microRNA profiles of exosomes, identifying specific miRNAs and associated pathways that play a part in the recovery of neurological function [113].

Investigations suggest that microglia play a crucial and diverse role in various neurological disorders, resulting in long-term neuronal damage. Notably, the M2-phenotype of microglia has been observed to activate protective mechanisms that prevent inflammation-induced neuronal death. Research has demonstrated that exosomal miR-137 derived from M2-phenotype microglia can inhibit neuronal death by targeting the Notch1 signaling pathway, which has been implicated in ischemia-induced injury [114]. Ubiquitin-specific protease 14 (USP14) has been identified as a pivotal contributor to brain damage resulting from ischemic stroke. However, recent research has demonstrated that exosomes released by M2 microglia containing miR-124 can reverse the detrimental effects of stroke and promote neuronal survival. These research findings provide evidence that exosomal miR-124 precisely directs its attention to and suppresses USP14 [115].

Exosomes sourced from MSCs in bone marrow (BMSCs-Exo) carrying long non-coding RNA ZFAS1 increased the level of SOD, reduced apoptosis, and suppressed inflammation in ischemic stroke. This effect was found to be due to the inhibitory effect of ZFAS1 on microRNA-15a-5p [116]. Early growth response 2 (Egr2) regulates pro-inflammatory cytokines and lessens the impact of ischemic stroke. BMSC-derived exosomes could reverse cerebral injury caused by MCAO/R by transferring Egr2 and stimulating the expression of sirtuin (SIRT)6, suppressing the Notch signaling [117].

It is well-known that circRNAs play a role in modulating autophagy. Exosomes containing circSHOC2 had a neuroprotective effect and reduced neuronal apoptosis caused by OGD by activating autophagy. This was achieved by inhibiting the activity of miR-7670-3p, which led to the increased expression of SIRT1 and prevented apoptosis. Therefore, the circSHOC2/miR-7670-3p/SIRT1 pathway shows promise as a target for alleviating ischemic stroke [118]. Yang et al. revealed that exosomes carrying circ-Rps5 improved acute ischemic stroke, improved cognitive deficits, and triggered a shift to M2 microglia. It was also observed that the upregulation of miR-124-3 and downregulation of SIRT7 counteracted the effect of circ-Rps5 on M2 microglial polarization [119].

Exosomes derived from astrocytes contain microRNA-34c, which inhibits the TLR7 pathway. This suppresses the NF-κB/MAPK signaling pathway, reduces inflammation, and alleviates neurological injury caused by I/R injury [120]. In another study, exosomes obtained from ADSCs transfer miR-30d-5p had a neuroprotective effect against cerebral injury. These exosomes also reduce inflammation, reverse the M1 microglial polarization phenotype associated with autophagy, and enhance M2 microglial polarization. Furthermore, the exosomal miR-30d-5p could significantly suppress Beclin-1 and Atg5 expression by targeting the 3’UTR of Beclin-1 and Atg5 [121]. In individuals suffering from acute ischemic stroke, the concentration of miR-126 in plasma is decreased. However, intravenous administration of exosomes facilitates the transfer of miR-126 after stroke, leading to improved functional recovery, neurogenesis promotion, microglial activation inhibition, and neuroinflammation reduction [122].

Exosomes decorated with a RAGE-binding peptid carrying an anti-miR-181a oligonucleotide were designed. Intranasal administration of these exosomes to a rodent model simulating MCAO inhibited TNF-α expression, enhanced Bcl-2 expression, and reduced the infarct size. Furthermore, targeted exosomes delivered anti-miR-181a oligonucleotide more efficiently than unmodified exosomes or naked ones [123]. Exosomes, through the transfer of miR-17-92, have been found to have a pivotal role in enhancing stroke recovery and promoting the restructuring of axon-myelin. This transfer mechanism results in the restraint of PTEN, ultimately stimulating the PI3K/Akt/mTOR pathway [124].

Altogether, exosomes have shown promising therapeutic effects in alleviating ischemic stroke. Further in vivo investigations are necessary to elucidate the exact mechanism of exosomes in alleviating ischemic stroke. In addition, engineering strategies to chemically or genetically modify exosomes surface can be applied to achieve targeted delivery (See Tables 1, 2, 3, 4 and 5).

**Table 1:** Potential therapeutic effects and mechanisms of action of exosomes and their cargoes in ischemic stroke treatment

Origin of exosomes	Exosomes content	In-vitro	In-vivo	Outcomes	Ref
astrocyte-derived exosomes	-	mouse hippocampal neuronal cell line HT-22	Male C57BL/6 mice	↓autophagy ↓apoptosis	[85]
Vascular endothelial cell	-	Mouse cortex NPCs	Male SD rat	↓apoptosis ↑BrdU/nestin-positive cells	[86]
BECs	-	primary mouse neurons	C57BL/6J mice	↓apoptosis ↑synaptic length ↑neural motor behavior	[88]
TSG101-Overexpressing Human Neural Stem Cells	-	N2A neuroblastoma cells	Male SD rats	↓Inflammatory cytokines ↓Infarction volume	[90]
human induced pluripotent stem cell	-	Embryonic cortical neurons of the rat	-	↑BDNF ↓PTEN ↑AKT	[92]
BDNF-Overexpressing HEK293	-	SY-5Y cells	SD rats	↓apoptosis ↓oxidative stress ↑mitochondrial membrane potential	[94]
human neural stem cell-derived exosomes	BDNF	Neural stem cells	Male SD rats	↑Tissue repair ↓Infarct volume	[95]
Plasma	Edaravone	HBMECs	Male SD rats	↑neurological performance, ↓ischemic damage	[97]
Macrophage	Edaravone	Mouse mononuclear macrophage leukemia cells (RAW264.7)	Male SD rats	↑M2 microglia polarization ↓ neuronal cells death	[98]
Macrophage	Baicalin	SH-SY5Y cells	SD rats	↓ROS ↑Nrf2/HO-1	[99]
Macrophage	Curcumin	(hCMEC/D3 cells	Male SD rats	↓inflammation ↓ROS ↓apoptosis	[100]
Blood of Sprague Dawley (SD) rats	Quercetin	SH-SY5Y cells	Male SD rats	↑Nrf2/HO-1 ↑neurological recovery	[101]
Astrocyte	Nampt	Primary neurons	Male C57BL/6 mice	↑autophagy	[102]
ADMSCs	PEDF	SY-5Y cells	-	↑autophagy ↓apoptosis	[104]
BMSC	Zeb2/Axin2	Primary cultured neuron	Male SD rats	↑Neurogenesis ↑Neural plasticity ↑BDNF	[106]
Mesenchymal stromal cell	miR-210	-	Male C57BL/6 mice	↑Angiogenesis	[108]
Bone Marrow MSCs	miR-21-5p	HUVECs	Male SD rats	↑Angiogenesis	[109]
ADMSCs	miR-22-3p	rat primary cortical neurons	SD rats	↓infarct volume ↓apoptosis ↓ KDM6B	[110]
Human urine-derived stem cells	miR-26a	neural stem cells	SD rats	↑Neurogenesis ↓ histone deacetylase 6	[111]
Human umbilical cord MSCs	miR-146a-5p	BV2 microglia	C57BL/6 mice	↓neuroinflammation ↓IRAK1/TRAF6	[112]
Rat plasma	-	-	SD rats	↓infarct volume ↑recovery of function ↓TLR4/NF-κB	[113]
M2 microglia	miR-137	primary cortical neurons	C57BL/6 mice	↓Notch1 ↓apoptosis ↓infarct volume	[114]
M2 microglia	miR-124	primary cortical neurons	Mice	↑neuronal survival ↓apoptosis ↓USP14	[115]
Bone marrow MSCs	long noncoding RNA ZFAS1	BV-2 cells	Male C57BL/6J mice	↑SOD ↓apoptosis ↓inflammation	[116]
BMSC	Egr2	N2a and bEnd.3 cells	C57BL/6 mice	↑SIRT6 ↓ Notch ↑angiogenesis	[117]
Ischemic-preconditioned astrocyte	circSHOC2	Astrocytes	Male C57BL/6J mice	↓apoptosis ↓miR-7670-3p ↑SIRT1	[118]
Hypoxic pre-treated ADSCs	circ-Rps5	-	Male C57BL/6 mice	↑M2 microglia polarization	[119]
Astrocyte	microRNA-34c	N2a cells	Male Wistar rats	↓NF-κB/MAPK ↓neurological damage	[120]
ADSCs	miR-30d-5p	primary microglia	Male SD	↑M2 Microglial Polarization ↓inflammatory cytokines ↓autophagy	[121]
ADSCs	miR-126	mouse BV2 microglial cells	Male SD rats	↑functional recovery ↑neurogenesis ↓neuroinflammation	[122]
-	anti-miR-181a oligonucleotide	Neuro2A	SD rats	↓TNF-α ↑Bcl-2 ↓infarct size	[123]
Multipotent mesenchymal stromal cells	miR-17-92	-	Adult male Wistar rats	↑functional recovery ↑axon-myelin remodeling ↑PI3K/Akt/mTOR ↓PTEN	[124]

BECs, Brain Microvascular Endothelial Cells; TSG101, Tumor susceptibility gene 101; BDNF, Brain-derived neurotrophic factor; PTEN, Phosphatase and tensin homolog; TLR, Toll-like receptor; TRAF, Tumor necrosis factor receptor-associated factor; IRAK, Interleukin-1 receptor-associated kinase; HBMECs, Human brain microvascular endothelial cells; ROS, Reactive oxygen species; Nrf2, Nuclear factor E2-related factor 2; hCMEC/D3 cells, Human brain microvascular endothelial cell line; ADMSCs, Adipose-derived mesenchymal stem cells; PEDF, Pigment epithelium-derived factor; BMSC, bone marrow-derived mesenchymal stromal cells; HUVECs, Human umbilical vein endothelial cells; MSCs, Mesenchymal stem cells; SOD, Superoxide dismutase; NF- κB, Nuclear factor- kappa B; MAPK, Mitogen-activated protein kinase; ADSCs, Adipose-derived stem cells; TNF- α, Tumor necrosis factor α; Bcl-2, B-cell lymphoma 2; Neuro2A, Mouse neuroblastoma cells

### Spinal cord injury (SCI)

According to the medical definition, SCI is characterized by an impairment of the spinal cord leading to neurological dysfunction, regardless of any disturbance in the spinal column [125]. Unfortunately, the current clinical management approach of surgical decompression does not appear to treat nerve damage effectively. Additionally, high-dose treatment with methylprednisolone is associated with various side effects. Thus, introducing a novel drug delivery system holds promise in providing a distinct and innovative approach [126]. Berberine is a crucial benzylisoquinoline alkaloid of quaternary nature that is present in various traditional medicine plants. It exerts anti-inflammatory effects by regulating multiple cellular physiological processes [127, 128]. Previous studies revealed that intranasal administration of berberine and cur-loaded transferosomes improved spatial memory in AD mice and increased antioxidant activity [129]. In a study by Gao and colleagues, exosomes derived from M2-type primary peritoneal macrophages were utilized as a delivery system for berberine. The precise delivery capability of these exosomes enables the focused administration of berberine to injury sites, resulting in an increased concentration at the desired location. Pharmacokinetic experiments revealed that the bioavailability of exosomes-berberine was 3.2 times higher compared to free berberine solution. Furthermore, exosomes-berberine reduced inflammatory and apoptotic markers, simultaneously increasing the M2 protein marker CD206 level while concurrently decreasing the M1 protein marker iNOS. These findings suggest that exosomes-berberine possesses properties that counteract inflammation and prevent apoptosis by promoting the shift from M1 to M2 macrophage/microglia polarization. Additionally, the study demonstrated that treatment with exosomes-berberine enhanced motor skills in mice with SCI, thereby highlighting its potential as an effective therapeutic agent for SCI [130]. Exosomes originating from olfactory unsheathing cells have been discovered to be taken up by microglia and have a suppressive effect on microglial inflammation. This outcome is attained through decreasing the expression of NF-κB and c-Jun signaling pathways. This study suggests that using exosomes for immunomodulation could be beneficial for repairing SCI [131]. Rutin, also known as sophorin, is a glycosylated antioxidant with potential for nerve regeneration [132]. To exploit this potential, a local drug delivery system was developed using Flos Sophorae Immaturus exosomes. The natural nano-sized rutin carriers embedded in a hydrogel. Interestingly, this formulation effectively improved motor dysfunction by reducing spinal inflammation and oxidative stress [133].

Exosomes obtained from primary M2 macrophages were modified to improve their stability. These modified exosomes, called Cur-Exo–nerve growth factor (NGF), demonstrated anti-inflammatory and neuroprotective effects. The amalgamation of exosomes and Cur significantly impacted the polarization status of M1 macrophages following damage, effectively mitigating the detrimental effects of uncontrolled inflammation on spinal cord tissue. This study also revealed the healing capabilities of Cur-Exos-NGF in facilitating the transportation of external NGF to the injury site, which resulted in a higher survival rate for damaged nerve cells [134]. In another investigation, using exosomes as carriers for resveratrol contributed to its improved solubility and enhanced ability to penetrate the BBB, leading to increased concentrations in the CNS. The administration of resveratrol-exosomes contributed to the restoration of neuronal function and the rehabilitation of paralyzed limbs. These positive effects were achieved by initiating autophagy and preventing neuron apoptosis by targeting the PI3K signaling pathway [135].

Similarly, exosomes originating from primary Schwann cells expedited the process of axonal remyelination by promoting autophagy and reducing apoptosis. This favorable outcome can be attributed to reduced EGFR expression [136]. Promoting angiogenesis has been demonstrated to accelerate recovery and regeneration following SCI. Therefore, increasing angiogenesis has potential as a therapeutic strategy for alleviating SCI. Exosomes obtained from human placenta MSCs have been found to possess angiogenic activity on endothelial cells and can restore neurological function in an SCI mice model [137]. These findings were further supported by Huang et al., who discovered that exosomes originating from M2 macrophages promoted functional recovery and induced angiogenesis after SCI. These effects are attributed to the induction of the HIF-1α/VEGF signaling axis [138].

FTY720 is an inhibitor of the sphingosine 1-phosphate receptor-1 (S1P1) and exhibits immune-modulating properties [139]. In a study, NSCs-exosomes were used as a carrier for FTY720. Research has demonstrated that FTY720-NSCs-exosomes possess therapeutic benefits for SCI by effectively controlling the PTEN/AKT pathway. The administration of FTY720-NSCs-Exos resulted in improved pathological changes, hindlimb function, apoptosis inhibition, and the alleviation of hypoxia in an experimental SCI model of mice [140]. The effectiveness of carriers such as exosomes can potentially be enhanced by incorporating specific substances, such as Angiopoietin Like 3 (ANGPTL3), known to stimulate the creation of new blood vessels, which play a vital role in SCI recovery [141, 142]. By combining exosomes from urine stem cells (USC-Exo) with ANGPTL3 in a hydrogel, researchers have discovered a promising therapeutic intervention for SCI recovery, as this innovative method significantly impacts angiogenesis. The PI3K/AKT signaling pathway governs the beneficial impacts of USC-Exo on angiogenesis [143].

To effectively treat acute SCI, the researchers utilized fibrin glue, an injectable hydrogel, to embed the exosomes. Impressively, this practical formulation showcased notable results in restoring nerve tissue by reducing inflammation and oxidative damage [144]. The study conducted by Jiang and his team revealed that exosomes sourced from neurons containing higher levels of miR-124-3p exhibited notable anti-inflammatory effects. This suppression of inflammation was achieved by initiating the (PI3K)/AKT/nuclear factor kappa B (NF-κB) signaling pathway, which has a pivotal function in regulating cell apoptosis and the release of inflammatory factors. Interestingly, it was also observed that exosomal miR-124-3p specifically targets myosin heavy chain 9 (MYH9), which eventually modulates the signaling mentioned above pathway and substantially inhibits inflammation [145].

Furthermore, the intravenous injection of exosomes miRNA derived from BMSCs has been demonstrated to possess the ability to induce polarization of M2 macrophage in rats. This effect is achieved by inhibiting Ern1, a stress sensor that regulates unfolded proteins, ultimately preventing cell apoptosis [146, 147]. In a separate study, Wei Liu and his team made an intriguing finding regarding the behavior of MSCs in hypoxic conditions. The researchers discovered that these MSCs release exosomes that contain higher levels of a molecule known as miR-216a-5p. Subsequent investigation uncovered that miR-216a-5p specifically targets a protein called TLR4. Notably, the administration of these exosomes led to the blockage of TLR4/NF-κB signaling and the stimulation of PI3K and AKT pathways [148]. Exosomes originating from MSCs have been discovered to increase miR-19b and miR-21 levels, reducing apoptosis by suppressing the manifestation of PTEN. It has been found that miR-19b and miR-21 directly target PTEN and impact neuronal cell death [149]. The intravenous administering of exosomes obtained from bone marrow MSCs elevated miR-181c expression and demonstrated the ability to effectively protect against SCI via suppressing PTEN expression and NF-κB signaling [150].

Instead of using traditional methods, an alternate approach involves delivering exosomes through the nasal passage using an intranasal delivery system. RNAi delivery faces obstacles such as nuclease degradation and failure to pass the BBB. Exosomes offer the potential to be used as a suitable carrier for RNAi. Intranasal administration of exosomes, originating from MSCs and containing PTEN siRNA, which can breach the BBB and effectively bind to the specific site of SCI. This treatment approach has shown positive outcomes in rat models featuring complete SCI, leading to decreased PTEN expression and subsequent regeneration of axons, formation of new blood vessels, and positive functional recovery. Additionally, this method successfully suppresses microglial activation, reducing neuronal inflammation. Moreover, a decrease in astrogliosis and microgliosis was observed. Overall, this noninvasive and safe approach can be regarded as a feasible treatment choice for SCI [151]. An important microRNA, miR-133b, is recognized for its pivotal role in neuronal differentiation and axon regeneration. Recent findings have unveiled that miR-133b directly targets RhoA, a member of the Ras homolog gene family [152]. The protective and regenerative effects of exosomes originated from MSCs, modified with miR-133b, were observed in rat models with SCI through systemic administration. This outcome is achieved by activating ERK1/2, STAT3, and c-AMP response element-binding protein (CREB) in vivo, which are recognized for their participation in neuronal survival while concurrently impending the expression of RhoA. Notably, exosomes delivering miR-133b upregulated GAP43, a protein crucial for axon regeneration [153]. Similarly, exosomes derived from microglia have shown comparable outcomes in promoting axonal regeneration and inhibiting neuronal apoptosis. These exosomes are enriched with miR-151-3p, which mediates the apoptotic-signaling pathway of p53, p21, and CDK1. Furthermore, the upregulation of p53 and p21 inhibits CDK1 expression [154].

miR-29b can regulate the expression of various proteins, including GAP43, NF200, and GFAP. These proteins are recognized for their vital role in facilitating the regeneration of neuronal cells. Through the modification of MSCs with miR-29b and subsequent intravenous administration of exosomes originating from these cellular sources, a significant augmentation in the count of neurons demonstrating positivity for GAP43 and NF200 was observed. Conversely, there was a decline in the quantity of neurons marked by GFAP-positivity. These promising outcomes suggest that this approach holds great promise for alleviating SCI [155].

Overexpressed miR-338-5p in exosomes originating from BMSCs was discovered to have a neuroprotective effect. This effect was observed through the upregulation of GAP43 and neurofilament-M expression and simultaneous downregulation of GFAP and myelin-related glycoproteins. It was found that miR-338-5p targeted the Cnr1 gene, leading to an increase in c-AMP levels. Consequently, cannabinoid receptor 1 (Cnr1) was inhibited, while receptor activator protein 1 (Rap1) was activated. By activating the PI3K/AKT biological route, cellular apoptosis was reduced, and the preservation of neuronal was promoted, all mediated by c-AMP-associated Rap1 induction [156].

The injection of exosomes derived from M1-BMDMs containing miR-155 to bEnd3 microvascular endothelial cells resulted in impaired motor function recovery in vivo. Additionally, it facilitated endothelial-to-mesenchymal transition (EndoMT), induced dysfunction relating to mitochondria, and elevated levels of ROS in vitro. These outcomes were linked to the stimulation of NF-κB by targeting SOCS6, effectively preventing p65 degradation via ubiquitination. This indicates that targeting the miR-155/SOCS6/p65 pathway could be beneficial as a therapeutic approach for SCI, but further investigation is necessary [157]. It is worth noting that miR-155 is also implicated in breast cancer and leukemia [158, 159]. On a related note, VGF has been found to play a role in myelination. Notably, VGF-enriched exosomes embedded in fibrin gel were found to promote oligodendrogenesis and subsequently improve SCI outcomes [160].

Collectively, exosomes could serve as hopeful therapies to combat SCI through the mitigation of several pathological pathways. Further investigations and innovative techniques are necessary to monitor the biodistribution of in vivo administered exosomes.

**Table 2:** Potential therapeutic effects and mechanisms of action of exosomes and their cargoes in SCI treatment

Origin of exosomes	Exosomes content	In-vitro	In-vivo	Outcomes	Ref
M2-type primary peritoneal macrophage	Berberine	bEnd.3 cells/ BV2 cells	Adult C57BL/6J mice	↓iNOS ↑CD206 ↓inflammatory and apoptotic cytokines (TNF-α, IL-1β, IL-6, Caspase 9, Caspase 8)	[130]
olfactory ensheathing cells	-	Primary neurons	Male SD rats	↓pro-inflammatory microglia polarization ↓NF-κB and c-Jun	[131]
*Flos Sophorae Immaturus*	Rutin	SHSY5Y cells	Female SD rats	↓spinal inflammatory ↓oxidative stress	[133]
primary M2 macrophages	Curcumin, nerve growth factor	PC12 cell	Adult male C56BL/6 mice	↓TNF-, IL-1, and IL-6, ↑ TGF-β	[134]
primary microglia	Resveratrol	Primary spinal cord neurons	Male SD rats	↑autophagy ↓apoptosis rehabilitation of paralyzed limbs	[135]
primary Schwann cells	-	PC12 cells	Female Wistar rats	↑autophagy ↓apoptosis ↑axonal remyelination ↓EGFR	[136]
Human placenta MSCs	-	HUVECs	Male mice	↑angiogenic activity ↑neurologic function	[137]
M2 macrophages	-	brain endothelial cell line (bEnd.3)	Adult SD rats	↑functional recovery ↑ angiogenesis ↑HIF-1α/VEGF	[138]
nerve stem cells	FTY720	SCMECs	SD rats	↓apoptosis amelioration of the hindlimb function ↓hypoxia	[140]
human urine stem cells	ANGPTL3	HUVECs	Mouse	↑angiogenesis	[143]
MSCs	-	-	Female SD rats	↓inflammatory ↓oxidative microenvironment	[144]
Neuron-conditioned medium	miR-124-3p	A1 astrocytes M1 microglia	Male mouse C57BL	↑functional behavioral recovery ↓Inflammation ↓M1 microglia ↑PI3K/AKT ↓ NF-κB	[145]
BMSCs-derived exosomes	miR-124-3p	Macrophage acquired from rats’ spinal cord	Male SD rats	↑M2 macrophage polarization ↓apoptosis ↓Ern1	[146]
Hypoxic and normoxic BMSCs-derived exosomes	miR-216a-5p	Microglia obtained from newborn mice brain	Male mice (C57BL/6)	↑functional behavioral recovery ↑M2 polarization ↓TLR4/NF-κB ↑ PI3K and AKT	[148]
MSCs-derived/ differentiated and undifferentiated PC12-derived exosomes	miR-21/miR-19b	SH-SY5Y/U251	Adult standard error rats	↓apoptosis ↓PTEN	[149]
BMSCs-derived exosomes	miR-181c	Rat microglia HAPI	Adult male SD rats	↓apoptosis ↓inflammation in microglia and spinal cord ↓PTEN ↓NF-κB	[150]
Human BMSCs (lonza)-derived exosomes	PTEN siRNA	-	Adult female SD rats	↑functional recovery in complete SCI rats/Axonal growth and neovascularization/ Reduced microgliosis	[151]
MSCs	miR-133b	-	Adult male SD rats	↑ERK1/2, ↑STAT3 ↑CREB ↓ RhoA ↑GAP43	[153]
Microglia	miR-151-3p	Primary cortical neurons isolated from fatal mice (E14-16)	Adult female C57BL/6 mice	↑functional recovery and axonal regrowth ↓neuronal apoptosis	[154]
BMSCs-derived exosomes	miR-29b	-	female SD rats	↑NF200 and GAP-43 ↓ GFAP	[155]
BMSCs	miR-338-5p	Rat pheochromocytoma PC12 cells and human embryonic kidney 293 HEK293	Male SD rats	↑GAP43 ↑neurofilament M ↓GFAP ↑ cAMP ↓Cnr1↑Rap1 ↓apoptosis	[156]
M1-polarized bone marrow-derived macrophages	miR-155	bEnd.3	Male mice C57BL/6	Mitochondrial dysfunction ↑ROS ↓motor function recovery	[157]
Rat BMSCs	VGF	-	Female C57BL/6 mice	↑oligodendrogenesis	[160]

iNOS, Inducible nitric oxide synthase; TNF- α, Tumor necrosis factor α; IL, interleukin; NF- κB, Nuclear factor- kappa B; TGF-β, Transforming growth factor β; EGFR, Epidermal growth factor receptor; VEGF, Vascular endothelial growth factor; HUVECs, Human umbilical vein endothelial cells; SCMECs, Spinal cord microvascular endothelial cells; PI3K/AKT, Phosphatidylinositol-3 kinase/AKT; PTEN, Phosphatase and tensin homolog; MSCs, Mesenchymal stem cells; STAT.Signal transducer and activator of transcription; CREB. c-AMP response element-binding protein; ERK, Extracellular-signal-regulated kinase; GAP43, Growth associated protein 43; GFAP, Glial fibrillary acidic protein; Cnr1, Cannabinoid receptor 1; Rap1, Receptor activator protein 1; ROS, Reactive oxygen species; VGF, Vascular growth factor; BMSCs, bone marrow-derived mesenchymal stromal cells

### Multiple sclerosis (MS)

MS is a neurological condition defined by the immune system attacking the protective myelin sheath surrounding nerve fibers, which can result in significant impairment [161]. This impairment can affect various parts of the neural system, resulting in physiological, cognitive, and occasionally psychological complications. The symptoms may vary depending on the nerves affected and the extent of nerve damage [162]. In research conducted by Wu et al., exosomes were extracted from the NSC of mice and modified with a lentivirus armed PDGFR ligand to create targeted exosomes. These targeted exosomes were used to deliver Bryostatin-1, which enhanced the myelin sheath’s protective function and facilitated remyelination. Furthermore, it inhibited astrogliosis, axon impairment, and the activation of pro-inflammatory microglia [163]. Microglia have a vital impact on monitoring the CNS, and an imbalance in their M1/M2 phenotypes contributes to the advancement of MS [164].

In a preclinical model involving animals to stimulate MS, researchers used macrophage-derived exosomes as an endogenous carrier of transporting resveratrol to the CNS. This innovative method effectively reduced inflammation in the central and peripheral nervous system [165]. The development of MS involves activating a range of cytokines, including TNF-α and IL-12, which play a role in provoking the inflammatory reaction and causing harm to myelin, the protective covering of nerve fibers. Conversely, IL-10 and TGF-β have been known as possible therapeutic options for managing this disease [166]. It has been discovered that exosomes released by BMSCs can enhance scores reflecting neural behavior, decrease the invasion of inflammatory cells in the CNS, and alleviate demyelination. Furthermore, the study documented an increase in M2-associated cytokines (IL-10 and TGF-β as well as a reduction in M1-associated cytokines (TNF-α and IL-12) after the administration of exosomes [167]. Moreover, the administration of exosomes obtained from IFN γ-stimulated MSCs via intravenous delivery resulted in a reduction in demyelination and neuroinflammation while also promoting the population of regulatory T cells (Tregs) in the spinal cords of mice with EAE [168].

Aptamers, which are small RNA and DNA molecules, possess secondary or tertiary structures that enable them to selectively bind to proteins or other targets within cells [169]. The LJM-3064 aptamer has been extensively studied and utilized as a targeted ligand and therapy due to its strong affinity for myelin, as indicated in the literature [170]. An in vivo study observed that when LJM-3064 aptamers attached to exosomes were administered before exposure to a specific pathogen, the Th1 response was dampened while the Treg population increased [171]. The biodistribution of the targeted exosomes should be conducted to determine the fate of the in vivo exosome. Exosomes engineered with the RVG peptide have shown a propensity for targeting the brain, offering potential improvements in drug delivery and efficacy for CNS disorders [172]. To further improve the transport of BDNF to the brain, Zhai et al. developed exosomes loaded with BDNF mRNA and modified with the RVG peptide. These modified exosomes were then administered via the nasal route to cuprizone mice, demonstrating the potential for highly effective delivery of BDNF, promotion of remyelination, and improvement in motor coordination [173]. Overall, exosomes, as both therapeutic and natural carriers, could combat MS, which is a promising therapy for attenuating MS through several mechanisms.

**Table 3:** Potential therapeutic effects and mechanisms of action of exosomes and their cargoes in MS treatment

Origin of exosomes	Exosome’s content	In-vitro	In-vivo	Outcomes	Ref
Mice neural stem cells	bryostatin-1	-	C57BL/6 male mice	↓astrogliosis ↓axon damage ↓pro-inflammatory microglia	[163]
macrophages (RAW-Exo)	Resveratrol	-	Female C57BL/6 J mice	↓inflammatory responses	[165]
BMSCs	-	HAPI microglia cell line	Male SD rats	↓infiltration of inflammatory cells into the CNS, ↓demyelination ↑IL-10 ↑TGF-β ↓TNF-α ↓IL-12	[167]
IFN γ-stimulated human MSCs	-	PBMCs	Female C57BL/6J mice	↓demyelination ↓neuroinflammation ↑Tregs	[168]
MSCs	LJM-3064 aptamers	OLN93	Female C57BL/6 mice	↓Th1 ↑Treg ↓inflammation	[171]
Genetic engineering of HEK293T cells	BDNF mRNA	OPCs	Male C57BL/6 mice	↑remyelination ↑motor coordination proficiency	[173]

BMSCs, Bone marrow-derived mesenchymal stromal cells; TNF- α, Tumor necrosis factor α; IL, Interleukin; CNS, Central nervous system; PBMCs, Human peripheral blood mononuclear cells; Tregs, Regulatory T cells; OLN93, oligodendroglia cell line; OPCs, Oligodendrocyte precursor cells; BDNF, Brain-derived neurotrophic factor

### Alzheimer’s disease (AD)

AD is marked by the buildup of Aβ plaques and hyperphosphorylated Tau in a particular brain region, resulting in synaptic deficits and neuronal loss [174]. The degradation clearance system involves several proteases that are crucial in breaking down extracellular Aβs. Despite, there is currently no treatment for AD, several management approaches have been explored [175, 176]. To address these issues, a study investigated a new therapeutic strategy for AD. The approach involved using macrophage-derived exosomes loaded with silibinin, a flavonoid compound. This approach aimed to preventAβ aggregation and enhance cognitive abilities in mice afflicted with AD.

The treatment with Exo-Slb (exosomes loaded with silibinin) inhibited astrocyte stimulation and decreased the release of various cytokines, including TNF-α, IL-6, and IL-1β. Furthermore, the treatment inhibited the NF-κB pathway, resulting in apoptosis blockage in Aβ-treated SH-SY5Y cells [177]. The naturally occurring flavonoid compound Que has demonstrated the potential to enhance cognitive function by protecting the brain, reducing oxidative stress, and decreasing inflammation. It may aid in mitigating Tau pathology, slowing down the formation of amyloid plaques, and inducing autophagy.

In a study involving mice afflicted with AD induced by okadaic acid (OA), the administration of Que-loaded plasma exosomes (Exo-Que) boosted the neuroprotective effects of Que. This was accomplished by inhibiting CDK5, a protein participating in Tau protein’s atypical phosphorylation. By inhibiting CDK5 activity, Exo-Que successfully reduced the development of insoluble neurofibrillary tangles (NFTs), representing a characteristic feature of AD. Furthermore, Exo-Que displayed enhanced anti-apoptotic effects by diminishing caspase 9 and caspase 3, proteins associated with cell death [178].

Cur-primed exosomes enhanced cognitive abilities in mice afflicted with OA-induced cognitive impairment. This improvement was attributed to the suppression of excessive phosphorylation of Tau protein by the AKT/GSK-3ß pathway and decreasing cell apoptosis. The significantly increased presence of Exo-Cur in the hippocampus was detected when exosomes derived from LFA-1 interacted with endothelial ICAM-1 to traverse the BBB [179].

CoQ10, a dietary supplement, possesses an anti-inflammatory and antioxidative stress effect. When Exo and CoQ10 were given together, they enhanced cognition function in AD induced by streptozotocin. This improvement was achieved by increasing levels of BDNF and sex-determining region Y-box 2 (SOX2) in the hippocampus [180].

A study discovered that when BMSC-exosomes were introduced via the lateral ventricle, they exhibited effectiveness, whereas injection through the caudal vein did not yield the same results. This intervention led to notable improvements in AD-like behaviors, suppression of microglial activation and brain inflammation, reduction of amyloid accumulation, the restoration of nerve cells, and increased expression of BDNF [181]. A search conducted by Chen and colleagues has discovered that MSC-exosomes offer therapeutic benefits for individuals with AD. These exosomes can break down Aβ plaques, improve cerebral glucose metabolism and cognitive performance, and regulate epigenetics and genetic expression. This potential treatment is particularly promising because it can be administered without intact cells. By downregulating HDAC4 expression, MSC-exosomes facilitate the restoration of target genes. Among the MSC-exosomes derived from Wharton’s jelly MSCs (WJ-MSCs), miR-29a is the most prevalent miRNA. This specific miRNA targets HDAC4, an enzyme crucial in histone modification that is abundantly present in the brain. HDAC4 is vital in maintaining homeostasis and regulating genes relevant to synaptic plasticity, neuronal survival, and neuron development. Notably, individuals with AD exhibit significantly elevated levels of nuclear HDAC4. Additionally, in an AD mouse model, the total level of HDAC4 (both cytoplasmic and nuclear) is elevated [175].

Genetic engineering techniques were employed to further enhance the therapeutic properties of exosomes and create a modified exosome composition with RVG peptide on its surface. This modification enabled the selective targeting of a7-nAChR, leading to enriched levels of a neprilysin variant that effectively degraded Aβ. The exosomes utilized in the research were sourced from stem cells originating from adipose tissue. Moreover, the RVG-EXO formulation was specifically designed to target the brain’s hippocampus. Administration of this formulation demonstrated a decrease in the expression of IL1a, TNF-α, and NF-ĸB while increasing the expression of the IL10. Combining EXO-RVG with CD10dm further improved therapeutic efficacy and reduced the presence of Aβ40 within N2a cells. The ultimate purpose was to develop a precise and potent exosome composition to manage AD effectively [82].

A separate investigation demonstrated that modifying exosomes originating from MSCs with the CNS-specific peptide RVG enhances cognitive abilities in APP/PS1 transgenic mice. The connection between MSC-Exo and RVG was achieved via a DOPE-NHS linker. Intravenous administration of MSC-RVG-Exo led to a noticeable decrease in plaque deposition and Aβ concentration and a significant decline in astrocyte activation. This administration also led to a noticeable reduction in inflammatory cytokine concentration [182].

Another investigation demonstrated the efficacy of utilizing miR-22-loaded exosomes derived from ADMSCs to prevent the onset of pyroptosis and the subsequent emission of inflammatory mediators by controlling gasdermin D (GSDMD) in the AD mice model. Exo-miR-22 inhibited pyroptosis, a programmed form of apoptosis dependent on inflammatory aspartase. This inhibition was associated with the diminished presence of CD206 and IBA-1, which indicates the suppression of microglia activation by Exo-miR-22. Furthermore, Exo-miR-22 inhibited the expression of GSDMD and p30-GSDMD and decreased the expression of NLRP3 and Caspase-1 [183]. Introducing an enhanced environment (EE) has effectively prevented cognitive damage in AD mouse models. This is achieved through various mechanisms, including reducing inflammation in astrocytes, increasing synaptic connections, and stimulating CP cells in the subiculum area of the hippocampus. Moreover, the implementation of EE has been shown to elevate the concentration of Th1-derived INF-γ in the bloodstream, which may impact the release of neurotrophic factors by the choroid plexus cells. These cells located in the cerebral plexus have been identified as a source of miR-146a exosomes that are released into the CSF [184].

Therefore, the results suggested that exosomes might be a hopeful natural agent in ameliorating AD by affecting several signaling pathways with their cargoes. Furthermore, more in-depth preclinical investigations are necessary to explain the exact mechanism of exosomes in AD and cause hope for clinical remedy of AD.

**Table 4:** Potential therapeutic effects and mechanisms of action of exosomes and their cargoes in AD treatment

Origin of exosomes	Exosomes content	In-vitro	In-vivo	Outcomes	Ref
Murine macrophage RAW264.7 cell line	Silibinin	SH-SY5Y cells	Male SD rats C57BL/6 mice	↓${\text{A}{\beta }}_{1-42}$ ↓inflammatory cytokines ↓NF-κB pathway	[177]
Plasma	Quercetin	-	C57BL/6 mice	↓ CDK5 ↓Tau ↓NFTs ↑cognitive function	[178]
curcumin-treated (primed) macrophage	Curcumin	brain microvascular endothelial cell line(hCMEC/D3)	C57BL/6 mice	↓phosphorylation of Tau ↑cognitive function	[179]
ADSCs-Exo	CoQ10	-	Wistar rats	improve cognition and memory deficiency ↑BDNF ↑SOX2	[180]
BMSCs	-	-	Male C57BL/6 mice	improve AD-like behaviors ↓IL-1ß, IL-6, TNF-a, Aß (1–42), p-Tau ↑synapse-related proteins ↑BDNF	[181]
WJ-MSCs	-	The human neuroblastoma cell line SH-SY5Y	J20 AD transgenic mouse	↓Aβ plaques, Improvement of brain glucose metabolism/cognitive function ↓HDAC4	[175]
ADSCs	Neprilysin	N2a cells	BALB/c mice	↓proinflammatory genes, IL1a, TNF-α and NF-ĸB, ↑IL10	[82]
MSCs	-	-	APP/PS1 double transgenic mice	↓ TNF-$\alpha$, IL-β and IL-6 ↑ of IL-10, IL-4 and IL-13	[182]
ADMSCs	miR-22	PC12 cells	APP/PS1 double transgenic mice	↓GSDMD ↓p30-GSDMD ↓NLRP3 ↓Caspase-1 ↓inflammatory factors such as IL-6, IL-1β and TNF-α.	[183]
choroid plexus	miR-146a	cultured CP cells	FAD-mice	↓NF-ĸB	[184]

NF- κB, Nuclear factor- kappa B; CDK5, Cyclin Dependent Kinase 5; BDNF, Brain-derived neurotrophic factor; SOX2, Sex-determining region Y-box 2; BMSCs, bone marrow-derived mesenchymal stromal cells; ADSCs, Adipose-derived stem cells; MSCs, Mesenchymal stem cells; WJ-MSCs, Wharton’s jelly MSCs; ADMSCs, Adipose-derived MSCs

### Parkinson’s disease (PD)

PD is recognized as the second most widespread neurodegenerative condition globally, impacting between 1% and 4% of individuals over the age of 60. The key clinical manifestations of PD encompass bradykinesia, muscle stiffness, tremors, and difficulty maintaining balance [185, 186]. This condition is distinguished by the deficiency of dopaminergic neurons and intracellular formations referred to as Lewy bodies (LB) in multiple brain areas [187]. These areas also exhibit neuroinflammation and clusters of α-syn gene mutation and duplication, which significantly contribute to the development of PD [185, 188].

**Table 5:** Potential therapeutic effects and mechanisms of action of exosomes and their cargoes in PD treatment

Origin of exosomes	Exosomes content	In-vitro	In-vivo	Outcomes	Ref
HEK293T	Aptamer F5R2	Neuro2A cell line	C57BL/6J female mice	↓α-syn aggregation ↓neurobehavioral defects in the PD mice model	[189]
BMSCs	ASO4	primary neuronal cultures derived from the α-syn A53T mice	Male transgenic mice expressing A53T human α-syn	↓α-syn aggregation. ↓degeneration of dopaminergic neurons ↑motor performance	[190]
Mouse blood	Dopamine	bEnd.3 cells	Kunming mice	↑Brain distribution of dopamine ↑Therapeutic effect of dopamine	[191]
Bovine milk	Epicatechin gallate	SHSY5Y cells	-	↓Mitophagy ↓apoptosis	[193]
Immature dendritic cell	Curcumin siSNCA	SH-SY5Y cells	C57BL/6 mice	↓α-syn aggregation	[194]
Blood	-	-	Male C57BL/6 mice	↑dopaminergic neurons ↓oxidative stress ↓neuroinflammation ↓ apoptosis	[195]
MSCs	-	HBMECs	Male BALB/c mice	↑angiogenesis ↑ICAM1-SMAD3/P38MAPK	[196]
normal astrocytes	miR-200a-3p	SH-SY5Y cells and primary mesencephalic dopaminergic neuron cultures	C57BL/6J mice	↓MKK4	[197]
ADSCs	miR-188-3p	MN9D mouse cells	C57BL/6J male mice	↓autophagy ↓pyroptosis ↑proliferation ↓CDK5 ↓NLRP3	[198]
HEK-293T cells	catalase mRNA	Neuro2A cells	C57BL6/J mice	↓neurotoxicity ↓inflammation	[199]
primary dendritic cells transfected with RVG-Lamp2b	Anti- α-syn shRNA-MC	human SH-SY5Y neuroblastoma cell line	Normal C57BL6/C3H F1 mice	↓ α-syn aggregation ↑dopaminergic neurons	[200]
BMSCs	Wnt5a	primary microglia and primary neuron co-culture	SD rats	↓neuronal inflammation ↑dopaminergic nerves	[201]
BMSC	Wnt5	-	Wistar rats	↑PPARγ ↓circadian rhythm dysfunction	[202]
MSCs	-	-	(α-Syn) A53T transgenic mice	↓cognitive impairment ↑Wnt5a	[203]

α-syn, Alpha-synuclein ; MSCs, Mesenchymal stem cells; HBMECs, Human brain microvascular endothelial cells; MKK4, Mitogen-activated protein kinase kinase 4; ADSCs, Adipose-derived stem cells; CDK5, Cyclin dependent kinase 5; NLRP3, Nod-like receptor family pyrin domain containing 3; BMSCs, Bone marrow-derived mesenchymal stromal cells; PPARγ, Peroxisome proliferator activated receptor γ

Throughout various research studies, scholars have extensively explored the impact of artificially created DNA aptamers on the accumulation of α-syn, a protein linked to PD. The buildup of abnormal α-syn leads to increased oxidative stress and dysfunction of mitochondria, ultimately resulting in the degeneration of dopaminergic neurons. In one study, researchers found that attaching RVG onto exosomes’ exteriors allowed for specific neuron targeting and successful delivery of the aptamer. This delivery method significantly reduced the creation of abnormal aggregation caused by α-syn preformed fibrils (PFF) and consequently decreased neurobehavioral defects in a PD mouse model [189]. Another efficient and safe delivery method using exosomes involved antisense oligonucleotides (ASO4 sequence) to downregulate α-syn aggregation. The exo-ASO4 also reduced the deterioration of dopaminergic neurons, mitigated α-syn expression, and enhanced motor performance in a PD mouse model [190].

One potential treatment for PD involves the use of dopamine, but its effectiveness is limited by BBB, which hinders its access to the brain. To overcome this challenge, dopamine can be entrapped in nanoparticles, allowing for better BBB permeation. However, there are still several challenges associated with this method, and researchers need to explore alternative approaches for distributing dopamine in the brain.

In a study, researchers encapsulated dopamine within blood exosomes using the saturated solution incubation technique. Interestingly, these exosomes demonstrated the ability to cross the BBB with transferrin-transferin receptor interaction, significantly improving dopamine distribution in the brain including the striatum and substantia nigra by over 15 times. Furthermore, when administered intravenously in a PD mouse model, dopamine-encapsulated exosomes displayed lower toxicity and higher therapeutic efficacy than free dopamine. Dopamine-encapsulated exosomes increased dopaminergic neurogenesis, enhanced endogenous dopamine, tyrosine hydroxylase, and antioxidant enzymes, subsequently improving the neurobehavioral function in a mouse model of PD [191].

Catechins have shown potential neuroprotective effects in preclinical studies. Among catechins, one that stands out is epicatechin gallate (ECG), which has remarkable neuroprotective properties. ECG has been proven to safeguard SHSY5Y cells from harm induced by H_2_O_2_. However, its practical use is currently limited due to issues regarding stability and bioavailability. To overcome this challenge, exosomes, which have a pivotal function in different biological mechanisms and retain the therapeutic efficacy of drugs, offer a promising solution by enhancing brain delivery and distribution efficiency [192]. According to reports, the PINK1/parkin pathway regulates mitochondrial autophagy. A recent study has shown that ECG-Exo has the potential to shield SHSY5Y cells against oxidative stress and neurotoxicity by interfering with the PINK1/parkin pathway, thereby inhibiting mitophagy. Notably, ECG-Exo has also been found to reduce cell apoptosis by hindering caspase 3 and augmenting the Bcl-2/BAX ratio [193]. To further enhance the therapeutic potential, scientists have developed a hybrid nanoparticle composed of a gene-chemo core (Cur/phenylboronic acid-poly (2-(dimethylamino)ethyl acrylate) nanoparticle) and a modified shell part with small RNA molecules that target SNCA, which is modified with RVG-exosome. This innovative nano-complex acts as a nano scavenger, effectively reducing α-syn aggregation and significantly improving motor performance in a PD mouse model [194].

PD is characterized by disruptions in neurotransmitter regulation, lipid and energy metabolism, and mitochondrial dysfunction, all of which can substantially impact the CNS. An in-depth analysis of exosomes obtained from serum and brain tissue has revealed notable differences in metabolite expression between PD and control mice. Treatment with blood exosomes has shown promising results in reducing nervous system inflammation and apoptosis in animal models of PD. Moreover, exosome therapy improves motor coordination and facilitates the restoration of lost dopaminergic neurons. Therefore, exosomes can serve as both a therapeutic intervention and a diagnostic tool for PD [195].

Exosomes originating from MSCs have been identified to alleviate PD symptoms induced by MPP + and boost the growth of human brain microvascular endothelial cells (HBMECs) by stimulating the ICAM1-SMAD3/P38MAPK signaling pathway [196]. It should be mentioned that developing biodistribution methods to find the fate of exosomes in animal bodies is an urgent need. Similarly, exosomes from normal astrocytes have demonstrated the ability to prevent apoptosis in SH-SY5Y cells and primary mesencephalic dopaminergic neuron cultures, thanks to the presence of miR-200a-3p, which inhibits the expression of MKK4 protein and mRNA [197]. The regulation of inflammasome/pyroptosis and autophagy was found to involve NAcht Leucine-repeat Protein 3 (NALP3) and CDK5, respectively. Introducing exosomes carrying miR-188-3p has demonstrated the ability to hinder pyroptosis by targeting NLRP3. Furthermore, miR-188-3p-enriched exosome suppressed CDK5-mediated autophagy and mitigated the levels of autophagy-related factors including LC3-II/I and p62 [198]. It has been well-established that lowered concentrations of redox enzymes, such as catalase and SOD, are closely linked to oxidative stress and neurodegeneration. The inclusion of catalase, a potent antioxidant, in exosomes facilitates its accumulation in the brain, resulting in neuroprotection and the reduction of inflammation. Regarding therapeutic intervention, delivering exosomal catalase mRNA using EXOtic devices has demonstrated the ability to decrease neurotoxicity in cellular and PD mice models [199].

PD is marked by the buildup of α-syn aggregates, making the reduction of α-syn a promising therapeutic target. While siRNA therapy is only effective in the short term, using shRNA-modified macrophages (shRNA-MCs) provides a long-lasting effect. Incorporating anti- α-syn shRNA-MC into exosomes modified with RVG has been found to suppress α-syn clumping and offset the deficit of dopaminergic neurons [200].

Recent reports have indicated that the disruption of the Wnt signaling pathway has a function in advancing PD and other NDs. It has been observed that during the differentiation of dopaminergic neurons, exosomes contain high levels of Wnt5a, which effectively reduces inflammation in the substantia nigra while protecting the dopaminergic nerves [201]. Moreover, the transfer of Wnt5 through exosomes has been shown to restore circadian rhythm disorder in a PD model by enhancing the function of peroxisome proliferation-activated receptor γ (PPARγ) [202].

In another study, exosomes isolated from BMSC could relieve cognitive disturbance in a PD model and modify the deregulated metabolism of neuron cholesterol by targeting the Wnt5a-receptor-related protein 1 (LRP1) signaling axis [203].

**Fig. 6:**
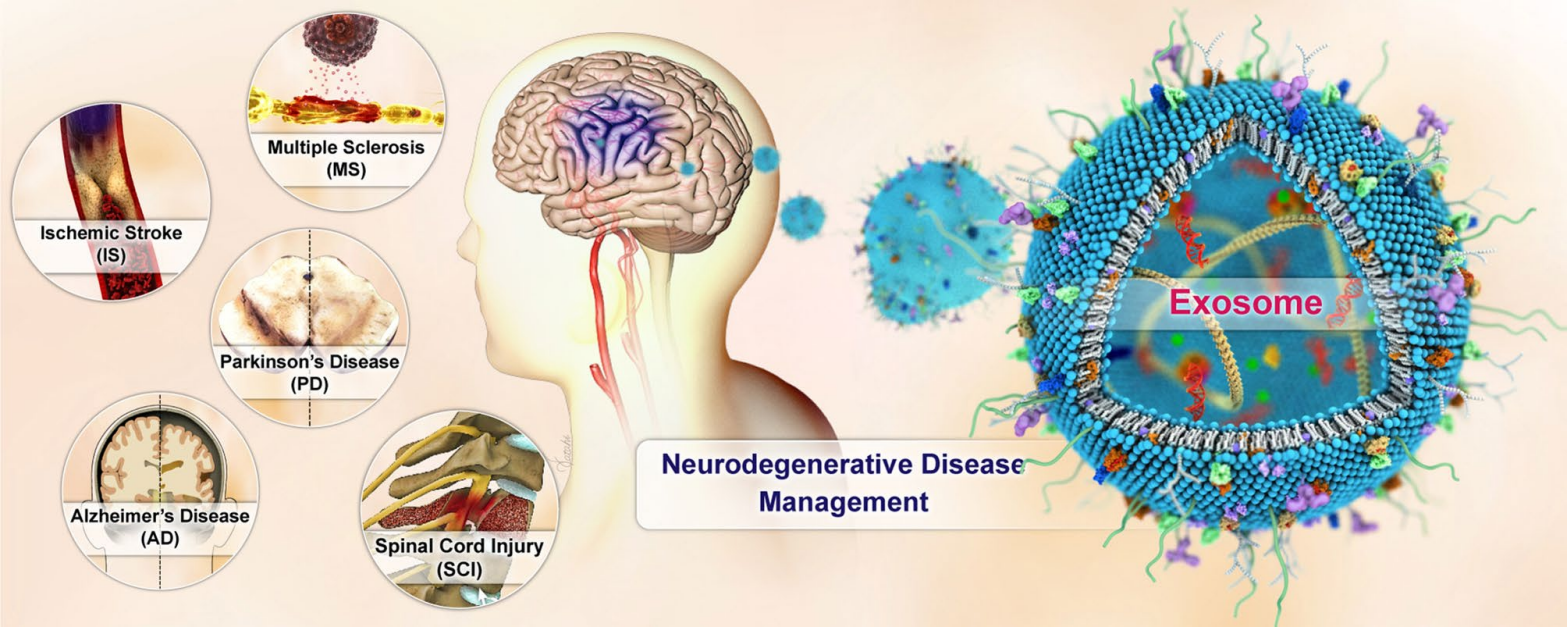
Therapeutic potential of exosomes in different types of NDs. Reprinted from Ref [8] with permission from Elsevier

Briefly, Exosomes can be a promising tool for delivering drugs, offering significant potential for over an extended period of drug availability and drug release, minimally invasive management of PD. Considering the absence of alternative efficient methods for transporting drugs to the brain, there is a rising demand for new approaches to prevent and treat neurological conditions. Exosomes provide a unique opportunity for efficient and regular drug delivery to the brain, establishing their significance as a crucial tool in alleviating several types of NDs (Fig. 6).

## Conclusion

The treatment methods of NDs are specially restricted by BBB. So, the use of therapeutic approaches to overcome the limitations is essential. Exosomes are nano-lipid bilayers released by nearly every cell in different biological fluids under physiological and pathophysiological conditions. Exosomes have several astonishing properties, making them suitable drug delivery systems for treating NDs. Various investigations have assessed the therapeutic potential of exosomes in several neurodegenerative diseases, such as AD, PD, MS, stroke, and SCI. These studies have demonstrated encouraging results that highlight the involvement of exosomes in alleviating of these conditions by boosting neurogenesis, hindering neuroinflammation, facilitating angiogenesis, and enhancing synaptic plasticity. While exosomes offer several unique properties for neurodegenerative therapy, the surface modification of exosomes is still required to increase targeted delivery, bypass the BBB, and achieve proper biodistribution. In addition, since exosomes transmit NDs misfolded proteins in various biological fluids, they can provide opportunities for early diagnosis, prognostic, and therapeutic intervention of NDs in the early stage. Many studies indicated that exosomes hold promise for the early detection and treatment of NDs, yet several unsolved issues exist. Some of these issues include: (1) improving engineered methods for increasing targeted delivery of exosomes, Engineering the surface proteins of exosomes allows for cell and tissue targeting specificity. Targeted delivery of neuroprotective agents by exosomes enhances the concentration of therapeutics in target cells and reduces potential side effects. (2) developing biodistribution methods for tracking the exosomes. The advancement of new techniques for exosomes’ imaging provide a more accurate depiction of exosomes’ distribution, enhance our understanding of exosomes’ involvement in normal and pathological conditions, and create a therapeutic approach based on exosomes that is approved for clinical use. (3) finding the exact BBB traversing mechanisms of exosomes. Significantly, exosomes can pass the BBB and control its physiological functions. However, the exat molecular mechanisms by which exosomes regulate the BBB is still not fully understood. (4) evaluation of beneficial effects and multiple action mechanism of exosome in the animal model. Further in vivo investigations are needed to fully clear the key benefits and mechanisms of exosomes in various NDs. The future of exosome-based therapy reveals great promise, but there are limitations that should be overcomed. These include addressing the challenges mentioned and solving regulatory assessments before introducing them into the clinical trials.

## Data Availability

No datasets were generated or analysed during the current study.

## Abbreviations

NDs: Neurodegenerative diseases
BBB: Blood-brain barrier
PD: Parkinson’s disease
AD: Alzheimer’s disease
MS: Multiple sclerosis
SCI: Spinal cord injury
MHC: Major histocompatibility complex
ICAM: Intercellular adhesion molecule
HSP: Heat shock proteins
ESCRT: Endosomal sorting complex required for transport
circRNAs: Circular RNAs
miRNAs: MicroRNAs
lncRNAs: Long non-coding RNAs
CD: Cluster of Differentiation
TSG101: Tumor susceptibility gene 101
GAPDH: Glyceraldehyde-3-phosphate dehydrogenase
TNF-β: Transforming growth factor β
TNF-α: Tumor necrosis factor α
pgk1: Phosphoglycerate kinase 1
Lamp: Lysosomal associated membrane protein
EGFR: Epidermal growth factor receptor
MSCs: Mesenchymal stem cells
ADSCs: Adipose-derived stem cells
Bcl-2: B-cell lymphoma 2
BNIP: BCL2/adenovirus E1B 19 kd-interacting protein
mTOR: Mammalian target of rapamycin
STAT: Signal transducer and activator of transcription
ULK: Unc-51 like autophagy activating kinase
VPS34: Vacuolar protein-sorting 34
ATG: Autophagy-related protein
LC3: Light chain 3
LPS: Lipopolysaccharide
PDCD4: Downregulating programmed cell death 4
PTEN: Phosphatase and tensin homolog
PI3K/AKT: Phosphatidylinositol-3 kinase/AKT
BAX: Bcl-2-associated X protein
Apaf-1: Apoptotic protease activating factor-1
FASL: Fas ligand
Mir: microRNA
IL: Interleukin
TGF-β: Transforming growth factor β
CCL: C-C motif chemokine ligand
NQO1: NAD(P)H quinone oxidoreductase 1
HO-1: Heme oxygenase 1
ROS: Reactive oxygen species
SOD: Superoxide dismutase
AMPK: (AMP)-activated protein kinase
Nrf2/ARE: Nuclear factor E2-related factor 2/ antioxidant response element
SIRT: Sirtuin
OPA1: Optic atrophy 1
ANT2: Adenine nucleotide translocator 2
GST: Glutathione S-transferase
SOCS: Suppressor of cytokine signaling
ERK: Extracellular-signal-regulated kinase
NF-Κb: Nuclear factor-κB
IRAK: Interleukin-1 receptor-associated kinase
TRAF: Tumor necrosis factor receptor-associated factor
TLR: Toll-like receptor
MyD88: Myeloid differentiation factor 88
TAK: Transforming Growth Factor-β-activated Kinase
NADPH: Nicotinamide adenine dinucleotide phosphate
IKK: Ikappa kinase
BCECs: Brain capillary endothelial cells
BDNF: Brain-derived neurotrophic factor
RMT: Receptor-mediated transcytosis
RVG: Rabies virus
AS: Astrocytes
OGD: Oxygen and glucose deprivation
MCAO/R: Middle cerebral artery occlusion/reperfusion
CNS: Central nervous system
EDV: Edaravone
PEDF: Pigment epithelium-derived factor
VEGF: Vascular endothelial growth factor
Ang-1: Angiopoietin-1
HDAC6: Histone deacetylase 6
NGF: Nerve growth factor
S1P1: Sphingosine 1-phosphate receptor-1
ANGPTL3: Angiopoietin Like 3
USCs: Urine stem cells
MYH9: Myosin heavy chain 9
Rap 1: Receptor activator protein 1
EndoMT: Endothelial-to-mesenchymal transition
GAP43: Growth associated protein 43
PEDF: Pigment epithelium-derived factor
CREB: c-AMP response element-binding protein
Slb: Silibinin
Que: Quercetin
Cur: Curcumin
NFTs: Neurofibrillary tangles
SOX2: Sex-determining region Y-box 2
WJ-MSCs: Wharton’s jelly MSCs
GSDMD: Gasdermin D
ASO4: Antisense oligonucleotides
ECG: Epicatechin gallate
LRP1: Receptor-related protein 1
